# A Novel Heterocyclic System Based on Natural Epoxyalantolactone

**DOI:** 10.3389/fchem.2019.00655

**Published:** 2019-10-01

**Authors:** Sergey G. Klochkov, Sergey A. Pukhov, Svetlana V. Afanasieva, Margarita E. Neganova, Ivan V. Ananiev, Marco Avila-Rodriguez, Vadim V. Tarasov, Gjumrakch Aliev

**Affiliations:** ^1^Institute of Physiologically Active Compounds of Russian Academy of Sciences, Chernogolovka, Russia; ^2^Institute of Organoelement Compounds of Russian Academy of Sciences, Moscow, Russia; ^3^Clinic Sciences Department, Health Sciences Faculty, University of Tolima, Ibagué, Colombia; ^4^Department of Pharmacology and Pharmacy, Sechenov First Moscow State Medical University (Sechenov University), Moscow, Russia; ^5^GALLY International Research Institute, San Antonio, TX, United States

**Keywords:** sesquiterpene lactones, epoxyalantolactone, synthesis, new heterocyclic system, Michael reaction

## Abstract

Natural sesquiterpene lactones which contain an exocyclic methylene group in the β-position of the lactone ring react readily with N-nucleophiles. When studying the reaction of the natural epoxyalantolactone with the primary amines we demonstrate the formation of a new heterocyclic system—the hydrogenated benzo[g]furo[4,3,2-cd]indol-3(1H)-one. Spectral data on the characteristics of the synthesized compounds are presented. The data on the reaction mechanisms and its applicability for the preparation are discussed.

## Introduction

Sesquiterpene lactones are a large group of plant-derived metabolites. The most studied sesquiterpene lactones were isolated from plants of the *Compositae* family. Among sesquiterpene lactones, a large number of compounds exhibit effective antitumor properties, with high specificity with respect to specific molecular targets (da Silva et al., 2018). Interestingly, sesquiterpene lactones were used as scaffolds for the synthesis of antitumor compounds (Grienke et al., 2018).

Natural sesquiterpene lactones containing the exocyclic methylene group in the β-position of the lactone cycle readily react with *N*-nucleophiles, including amines. Because of this transformation, other functional groups present in the molecule are usually retained with the subsequent formation of amino derivatives (Lawrence et al., 2001). These novel compounds possess promising biological activity (Hwang et al., 2006; Cantrell et al., 2010) and can be considered as potential candidates to act as drugs and pro-drugs (Woods et al., 2013).

We have found that the products of the reaction of epoxyalantolactone (**1**) with tryptamines are representatives of a new heterocyclic system: hydrogenated benzo[g]furo[4,3,2-cd]indol-3(1H)-one (Klochkov et al., 2012). They are formed due to the intramolecular nucleophilic substitution of the oxygen atom of the epoxide fragment with the amino group that resulted in recyclization: the oxirane cycle undergoes ring opening and the pyrrole ring undergoes ring closure. The present work is devoted to the detailed examination of this transformation.

## Result and Discussion

**Scheme 1:**
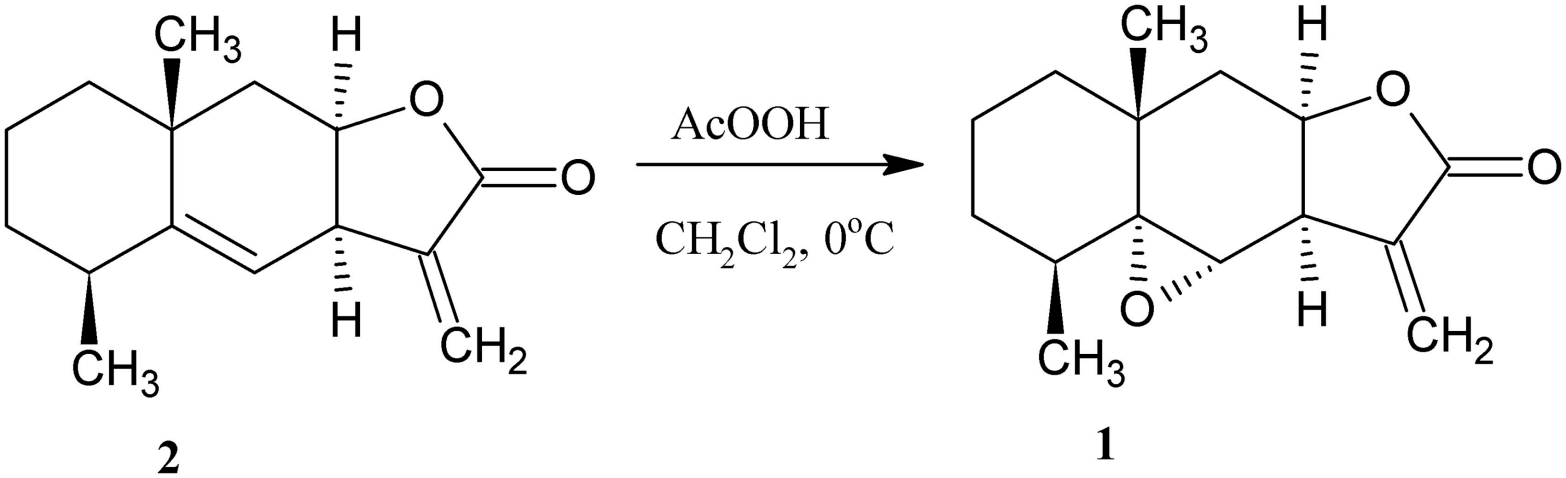
Synthesis of epoxyalantolactone **1**.

Epoxyalantolactone (**1**) is the minor secondary metabolite of plants belonging to the *Inula* family. The content of the natural epoxy derivative in plants usually does not exceed 0.01%. However, this derivative can easily be obtained by the semisynthetic method. For this purpose, alantolactone (**2**) was isolated from *Inula helenium* roots, and the endocyclic double bond in **2** was oxidized by peracetic acid (Klochkov et al., 2006) (Scheme 1).

Various primary amines were introduced into the reaction with lactone **1**. It turned out that the result of the reaction depended on the amine structure. Aromatic amines do not react with epoxyalantolactone, evidently, because of their lowered nucleophilicity, and simple aliphatic amines (as stronger bases) form complicated mixtures of compounds in which the products of lactone ring opening were detected by the ^1^H NMR method. Interestingly, the proper compounds were obtained using amines **3a–k** in the reaction with lactone **1**. Amines **3a–k** contain an additional n- or p-donating fragment (amino group, benzene, or indole cycle) separated from the amino group by the hydrocarbon bridge. Moreover, the presence of these groups increases the nucleophilicity of the nitrogen atom and favors heterocyclization to occur.

**Scheme 2:**
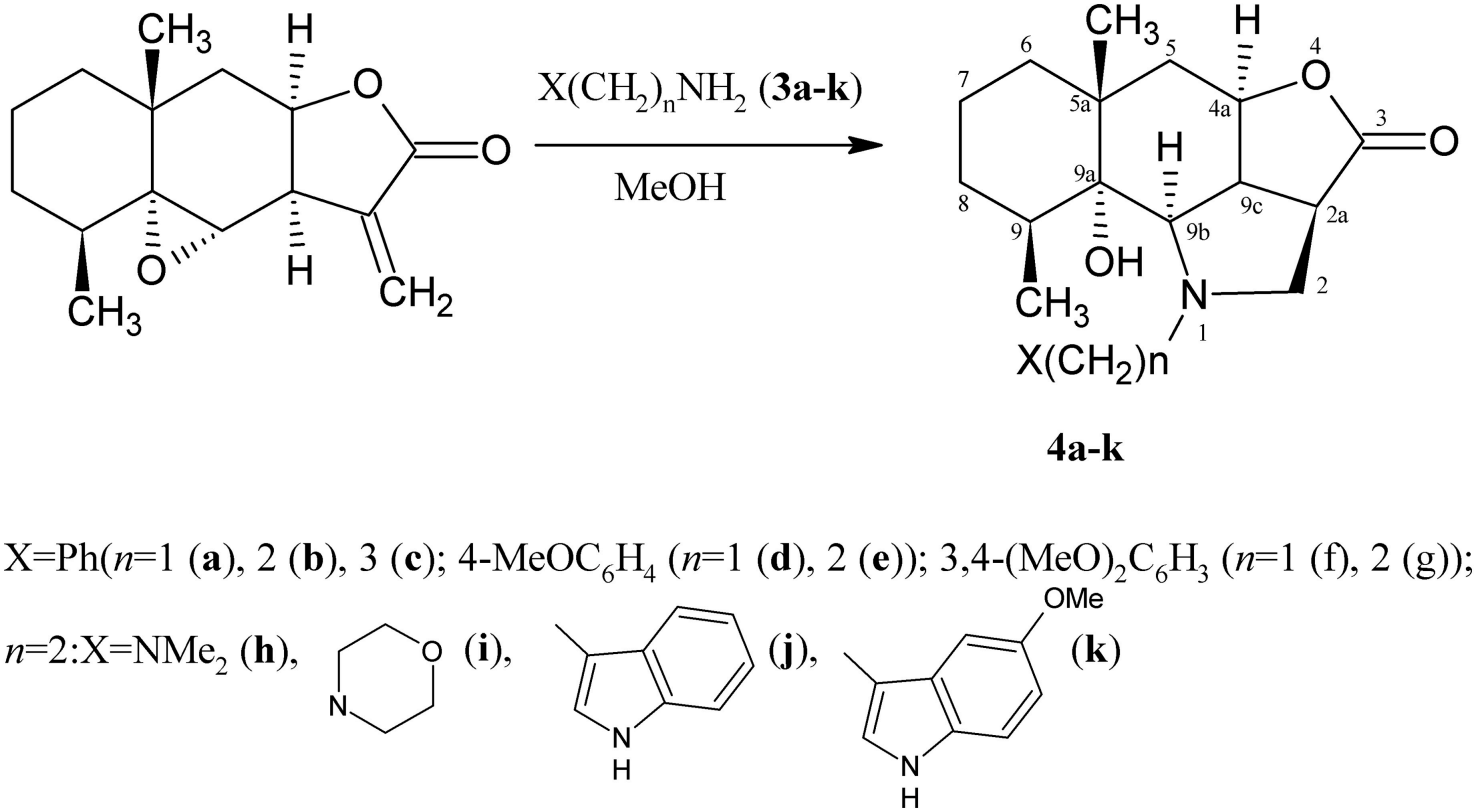
Synthesis of the derivatives **4a–k**.

Lactone **1** reacts with amines **3a–k** under mild conditions, *visualized* in methanol at ambient temperature in the presence of a small nucleophile excess, to form the corresponding hydrogenated benzo[g]furo[4,3,2-cd]indol-3(1H)-ones **4a–k** (Scheme 2).

As describe above, the process is stereospecific, since one spatial isomer is formed in all cases. This results in a new asymmetric center C-2a and configuration inversion at the carbon atom in position 9b. The structures of compounds **4a–k** were determined by spectroscopic methods (analysis of the IR, ^1^H and ^13^C NMR spectra including COSY and NOESY experiments).

As should be expected, the main signals from protons of the starting lactone are retained in the ^1^H NMR spectra of derivatives **4**, and the corresponding signals from protons of the amine fragment and protons at the C-2, C-2a, and C-9b atoms appear at 2.5–4.0 ppm, whereas the characteristic signals of the exocyclic = CH_2_ group (doublets at 5.82 and 6.46 ppm) are absent.

For example, the ^1^H NMR spectrum of compound **4b** exhibits distinct signals from protons at the C-2 atom as a doublet of doublets at 2.49 ppm and a doublet at 3.95 ppm having a high geminal spin-spin coupling (SSC) constant with each other (9.9 Hz) and vicinal interaction constant with the proton at the C-2a atom equal to 7.6 Hz. The signal from the 2a-H proton (3.21 ppm) appears as a multiplied doublet of doublets of doublets (ddd) for which the far-range interaction constant with the axial proton at the C-5 atom and the vicinal constant with the 9c-H proton (7.6 Hz) can be found. The signal from the 9b-H proton is presented by a pronounced doublet at 2.53 ppm having a SSC constant of 10.2 Hz with the 9c-H proton, which is manifested as a triplet of doublets at 3.39 ppm. The spectra also contain signals from protons of the NCH_2_CH_2_ ethylene bridge at 2.09 and 3.25 ppm and of NCH_2_C0048_2_ at 2.79 and 2.83 ppm, as well as resolved signals from aromatic protons: at the C-2′ and C-6′ atoms as a doublet of doublets at 7.17 ppm (*J*_1_ = 7.4, *J*_2_ = 1.4 Hz), at the C-4′ atom as a triplet of triplets at 7.21 ppm (*J*_1_ = 7.4, *J*_2_ = 1.4 Hz), and at the C-3′ and C-5′ atoms as a triplet of triplets at 7.29 ppm (*J*_1_ = 7.4, *J*_2_ = 1.4 Hz), which confirms the structure proposed for compound **4b**.

**Figure 1:**
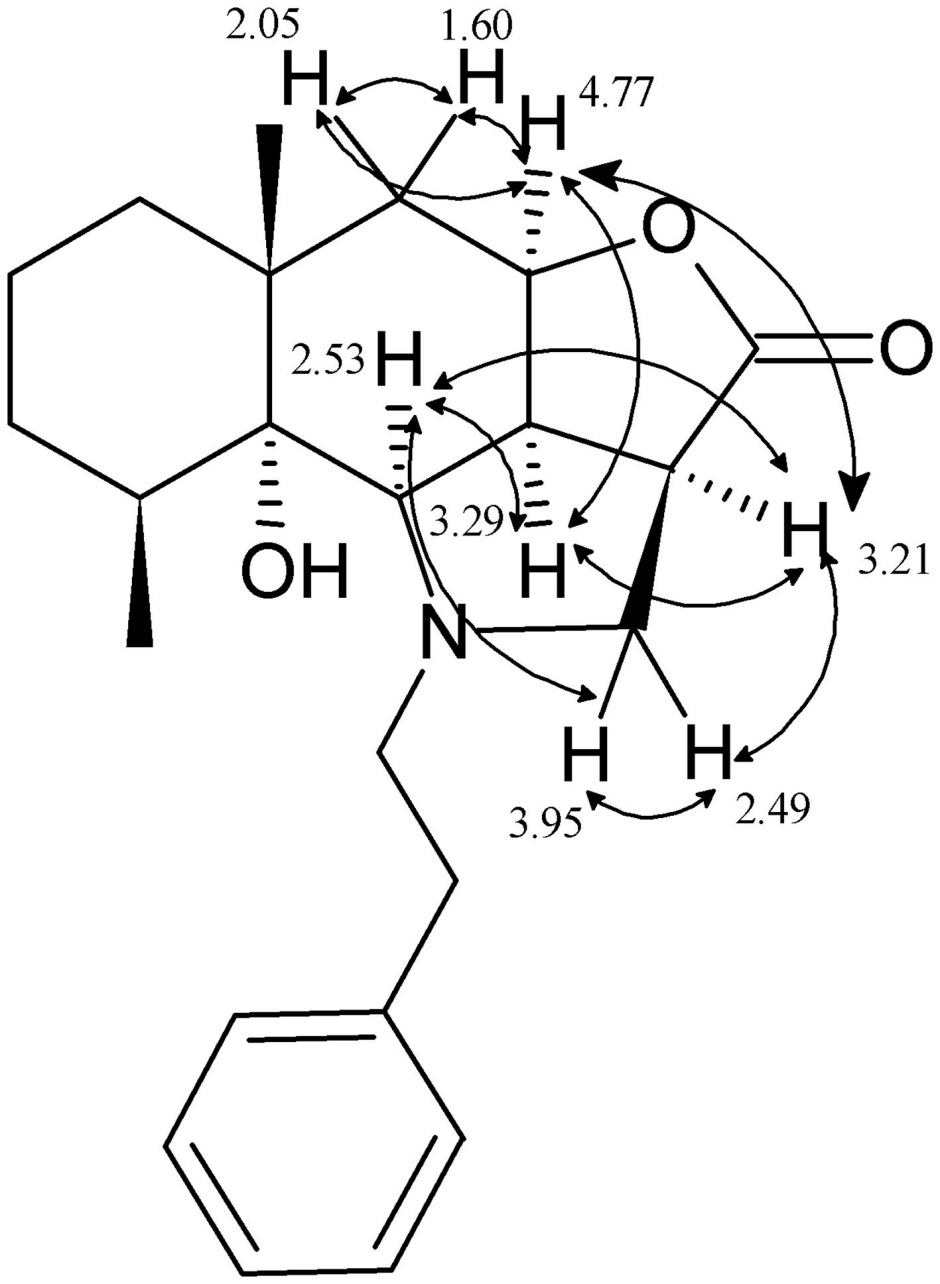
Structural significant NOE-correlations for **4b**.

Stereochemistry of the new asymmetric center of compounds **4a–k** at the C(2a) carbon atom was determined by proton analysis and correlation of vicinal interactions and NOE correlations in NOESY experiment. The main NOE correlations of the alicyclic fragment are presented in Figure 1 for compound **4b** as an example.

In an epoxyalantolactone (**1**) molecule, the proton in position 4a has the α-orientation as the proton at the C-9c atom, which is experimentally confirmed by a low spin-spin coupling constant (^3^*J*_3a−H, 9a−H_ = 7.3 Hz) and indicates the *cis*-conjunction of the lactone and decahydronaphthalene cycles. The NOESY spectrum of compound **4b** exhibits distinct NOE correlations between the protons 4a-H and 9c-H, 4a-H and 2a-H, 4a-H and 5-H_eq_, and 4a-H and 5-H_ax_. The proton at the C-9c atom is characterized by pronounced correlations between the proton at the C-2a atom and the proton at the C-9b atom appeared due to the addition of amine. Based on these data, we can conclude that the 2a-H, 4a-H, 9b-H, and 9c-H protons are arranged at one side of the hydrogenated naphtho[2,3-*b*]furan-2-one system; i.e., the newly formed asymmetric center has the β-configuration (stereodescriptor *R*). Similar regularities were revealed by an analysis of the spectra of other products **4**.

**Figure 2:**
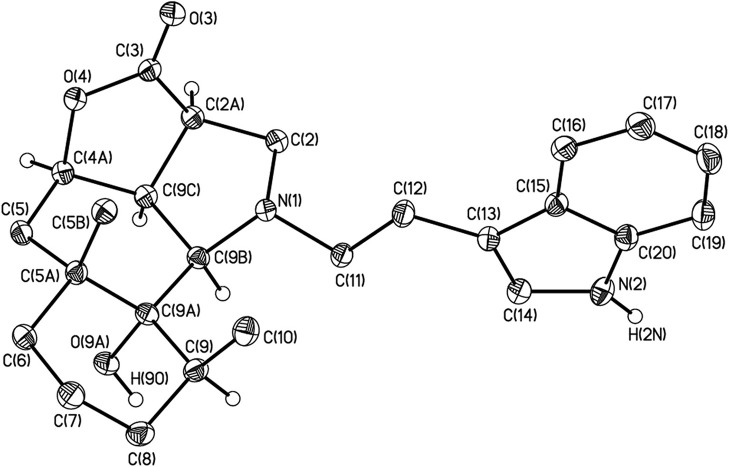
The general view of compound **4j** in crystal in the representation of the non-hydrogen atoms by probability ellipsoids of atomic displacements (*p* = 50%). For clarity the hydrogen atoms bounded to heteroatoms and tertiary carbon atoms are only shown.

**Scheme 3:**
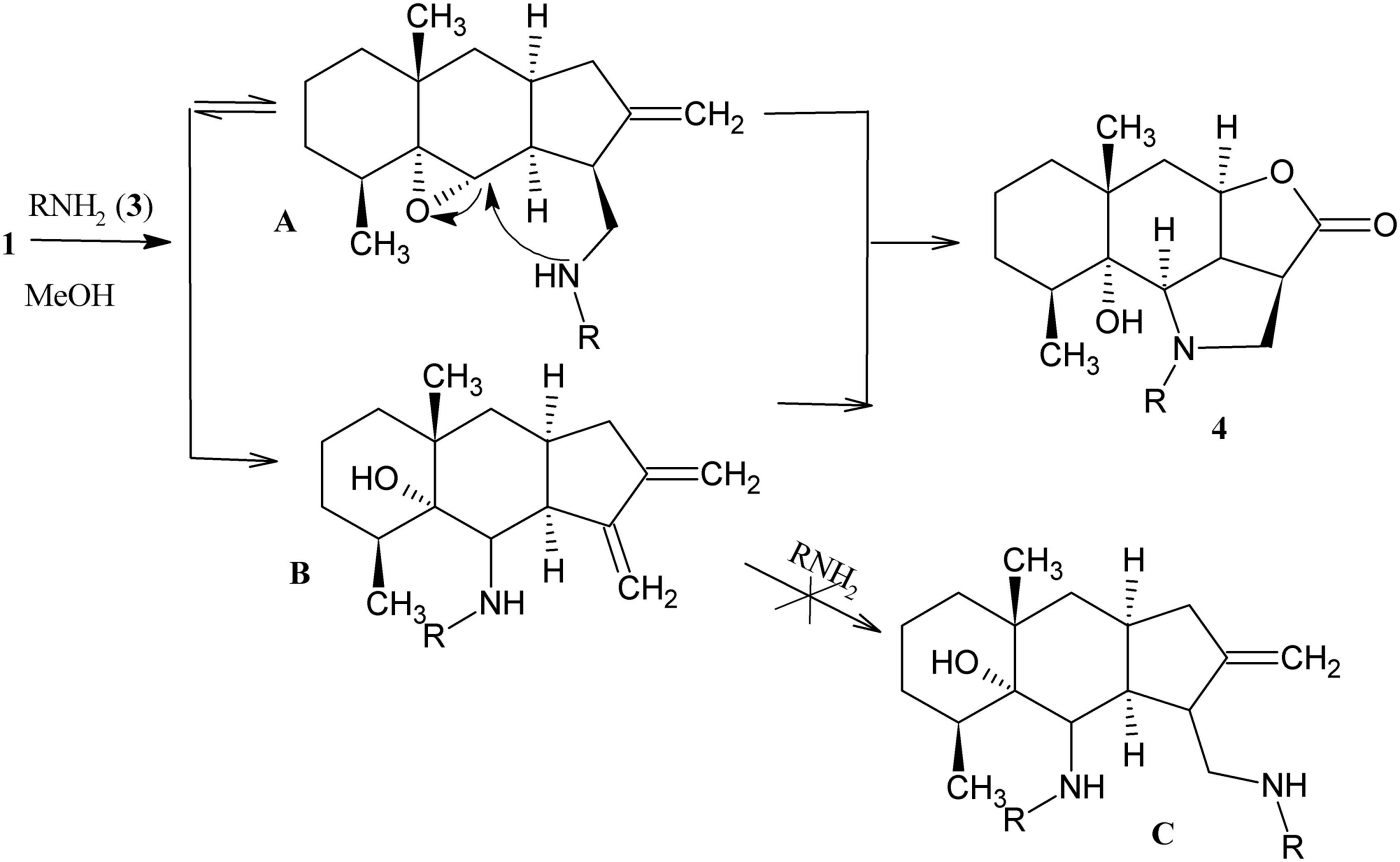
Mechanism of heterocyclization.

The structure of **4j** was confirmed by the single crystal X-ray diffraction method (Figure 2). According to this data, the crystal of **4j** showed both saturated carbon cycles. Also the 4j crystal displayed a chair conformation, as expected. Furthermore, the lactone and pyrrolidine cycles are characterized by envelope conformations with atoms C-2a and C-2 going out from the mean-square planes of C-3, O-4, C-4a, C-9c atoms, and N-1, C-9b, C-9c, C-2a atoms on 0.373(1) and 0.548(1) Å, respectively. All bond lengths and valence angles in **4j** have values being expected for this class of compounds (see, e.g., structure WAMFEX in Cambridge Structural Database) (Allen, 2002). The molecules of **4j** are bounded into layers by N-H…O and O-H…O hydrogen bonds (N…O 2.991(1) Å and O…O 2.793(1) Å). In turn there are only weak C-H…O, C-H…N and C-H…π contacts between these layers. Two routes of formation of hydrogenated benzo[*g*]furo[4,3,2-*cd*]indolones **4** are theoretically possible for the reaction of epoxyalantolactone (**1**) with primary amines (Scheme 3).

According to route *a*, at first the nucleophilic addition of amine occurs to the activated double bond of lactone (of the reversible Michaelis reaction type) and then the secondary amino group attacks the tertiary carbon atom *via* the S_N_2 nucleophilic substitution mechanism. Route *b* differs by the sequence of stages of amine addition. Both the theoretical factors and practical results indicate in favor of the predominant occurrence of the reaction *via* route *a*.

We suggest three possible explanations: first, the electrophilicity and steric accessibility for the nucleophilic attack of the trisubstituted oxirane fragment is lower than those of the activated methylene group. Second, a comparison of intermediates **A** and **B** shows that the intramolecular attack of the aminomethyl fragment to the epoxide ring is sterically preferable than the attack of the aminoalcohol fragment to the methylene group. Third, unsaturated aminoalcohols **B** and bis(amino) adducts **C** were not found in noticeable amounts in the reaction products, while the Michael adducts (intermediates **A**) were detected in the reaction mixtures, isolated, and characterized (see below).

Evidently, the nucleophilicity of the nitrogen atom in the initial primary amine is lower than that in the corresponding secondary amine (Michael adduct). For this reason, the rate of oxirane ring opening upon the attack of the amino group to the adduct is higher than that in the case of the initial amine, which also favors route *a* to occur.

**Scheme 4:**
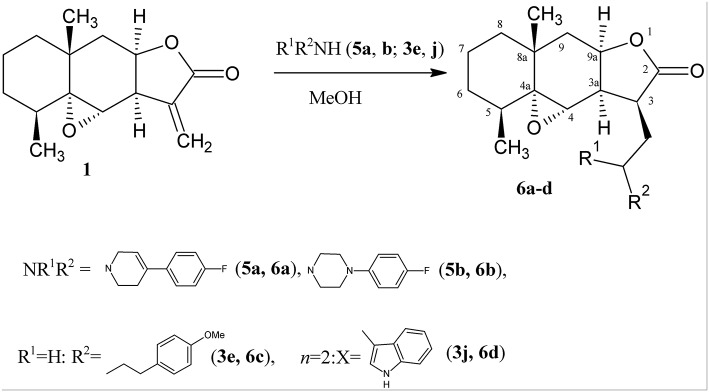
Synthesis of the aminodihydroalantolactone **6a–d**.

To confirm the proposed mechanism, we introduced secondary amines (4-fluorophenyl-substituted tetrahydropyridine and piperazine) into the reaction with lactone **1**. As a result, only the products of amine addition to the exocyclic double bond, namely, 4,4a-epoxy-3-aminomethyl-5,8a-dimethyldecahydro-naphtho[2,3-b]furan-2-ones **6a,b**, were obtained in good yields (Scheme 4).

In this case, the amino group becomes tertiary and the oxirane cycle is retained in the molecule. The formation of these derivatives indirectly indicates that heterocyclization proceeds *via* route *a* (see Scheme 3).

All 3-aminomethyldihydroalantolactones^1^ are also formed as one diastereomer. Their structures (including the configuration of the asymmetric center at the C-3 atom) were established by spectral methods. As in the ^1^H NMR spectra of derivatives **4a-k**, in the spectra of 3-aminomethyldihydroalantolactones **6a–d** the main signals from protons of the initial lactone are retained, the corresponding signals from protons at the C-3 atoms and 3-CH_2_ appear at 2.8–3.3 ppm, and the characteristic signals of the exocyclic = CH_2_ group are absent.

For example, the ^1^H NMR spectrum of compound **6a** exhibits pronounced signals from the 3-CH_2_ protons as a doublet of doublets at 2.93 and 2.99 ppm with a high geminal spin-spin coupling constant with each other (13.1 Hz), the vicinal interaction constant with the proton at the C-3 atom equal to 11.2 Hz, and an interaction constant of 4.0 Hz with the proton at the C-3a atom. The signal from the 3-H proton (3.26 ppm) appears as a distinct doublet of doublets for which the interaction constant with the proton at the C-9a atom equal to 7.3 Hz and the constant with the 3-CH_2_ proton (the signal of the corresponding proton in compounds **4a–k** represents a more complicated multiplet) can be found. The most significant distinctions in the spectra of the “closed” and “open” isomers are observed for the signals from the 3-CH_2_ protons. In compounds **4a–k**, the protons at the C-2 atom, which in the composition of the new cycle, correspond to these protons. For example, in the spectrum of compound **4b** these protons appear as a doublet of doublets at 2.49 ppm and a doublet at 3.95 ppm. Since they are arranged in the rigidly fixed cycle, their signals are shifted: the signal from the 2-H_β_ proton undergoes the most significant downfield shift (3.95 ppm) and additionally becomes a doublet. Compounds **4** and **6** can rather easily be distinguished by the presence or absence of this signal. The ^1^H NMR spectrum of compound **6a** also exhibits signals from protons of the amine moiety of the molecule. The well-resolved signals from the aromatic protons are observed: at the C-3″ and C-5″(symbol “corresponds to aromatic cycle) atoms, they appear as a triplet of triplets at 7.05 ppm (*J*_1_ = 8.8 Hz, *J*_2_ = 2.0 Hz) and at the C-2” atoms, they are observed as a doublet of doublets at 7.38 ppm (*J*_1_ = 8.8, *J*_2_ = 5.3 Hz). The spectrum also contains the signals from the piperidine fragment, namely, a triplet of the methylene H-3′ proton at 6.03 ppm, and the signals from protons at the C-2′ atom as a doublet of doublets at 3.15 and 3.37 ppm with a high geminal constant of 16.7 Hz with each other and with a vicinal constant of 3.2 Hz corresponding to the interaction with the 3′-H proton. The ^13^C NMR spectrum of compound **6a** exhibits the signal from the C-4″ atom as a doublet with the characteristic spin-spin coupling constant with the fluorine atom equal to 239.40 Hz.

**Figure 3:**
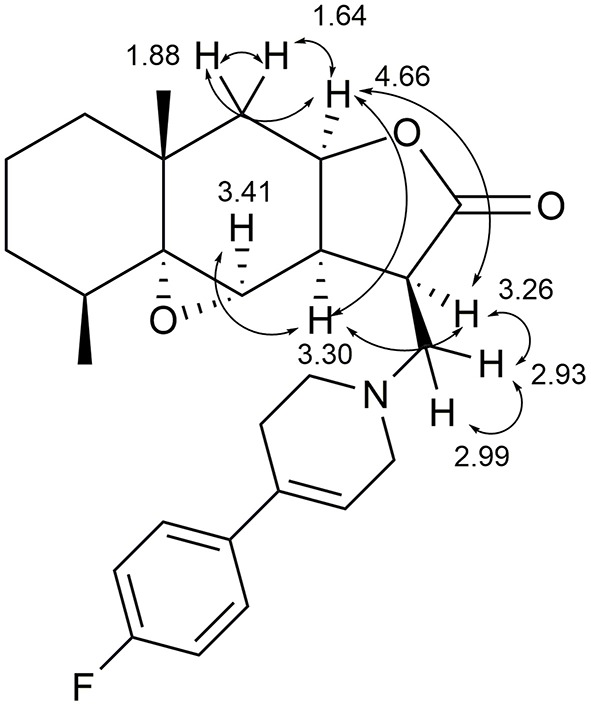
Structural significant NOE-correlations for **6a**.

Stereochemistry of the new asymmetric center at the C(3) carbon atom in compounds **6a–d** was determined from an analysis of proton correlations of vicinal interactions and NOE correlations in the NOESY experiment. The main NOE correlations of the alicyclic fragments are presented in Figure 3 for compound **6a** as an example.

The NOESY spectrum of compound **6a** exhibits the distinct NOE correlations between the 9a-H and 3a-H, 9a-H and 3-H, 9a-H and 9-H_eq_, and 9a-H and 9-H_ax_ protons. The proton at the C-3a atom is characterized by pronounced correlations with the α-oriented protons in position 4 and the proton at the C-3 atom formed after the addition of amine. Based on these data, it can be concluded that the 9a-H, 4a-H, 3a-H, and 3-H protons are localized at one side of the hydrogenated naphtho[2,3-*b*]furan-2-one system and the aminomethyl substituent has the β-configuration (stereodescriptor *R*) as for cyclic products **4**. Similar regularities were revealed by an analysis of the spectra of compound **6b**.

It should be mentioned that we obtained similar “*open”* products **6c, d** also in the reactions involving two primary amines: 2-(4-methoxyphenyl)ethylamine and tryptamine. So, the reaction of epoxyalantolactone (**1**) with 2-(4-methoxyphenyl)ethylamine (**3e**) affords a mixture of two compounds, which was separated by HPLC into heterocyclization product **4e** (yield 76%) and Michael adduct **6c** (yield 10%). It also turned out that the evaporation of the solvent (methanol) 0.5 h after mixing of lactone **1** with tryptamine (**3j**) followed by chromatography of the reaction mixture gave amino derivative **6d** in a yield of 70%. Heterocyclization products **4e**, **j** can easily be distinguished from the corresponding Michael adducts **6c, d** by signals from protons in the ^1^H NMR spectra. So, the signal from one of the protons at the C-2 atom, which is in the composition of the new cycle, undergoes the downfield shift by almost 1 ppm because of deshielding. These characteristic signals make it possible to perform NMR monitoring of the reaction and to control the formation of isomeric compounds.

The model experiment was carried out to prove the routes of formation of hydrogenated benzo[g]furo[4,3,2-cd]indol-3(1H)-ones **4**. Tryptamine derivative **6d** in deuterated chloroform was placed in a NMR tube of the spectrometer, and the ^1^H NMR spectra were recorded at certain time intervals. The NMR monitoring of the reaction showed that 24 h after dissolution the spectra contained signals from protons of the initial lactone **1** (the most informative region of signals of the = CH_2_ group). Its amount in the solution is ~18% indicating that the Michael addition reaction is reversible. Amino derivative **6d** is transformed with time into new heterocycle **4j**, which is indicated by the appearance of signals from the corresponding protons in the ^1^H NMR spectrum (the characteristic signal from the H-2β proton appears at 4.06 ppm as a doublet with a constant of 9.8 Hz). The complete disappearance of the signals from compound **6d** is observed in 120 h. It should be mentioned that upon keeping in methanol at ambient temperature Michael adduct **6d** undergoes cyclization within 1 day (yield 94%).

Thus, the representatives of the new heterocyclic system was obtained from natural alantolactone (**2**) by the reaction of intramolecular nucleophilic substitution. They are interesting as potent biologically active compounds and will be tested and validated subsequently in the corresponding activity models elsewhere.

## Experimental Section

### General

All chemicals and solvents were reagent grade and used without purification. IR spectra were recorded on a Bruker ZFS-113 instrument using KBr pellets. ^1^H and ^13^C NMR spectra were recorded on a Bruker Avance III instrument (500 and 125 MHz, respectively) using CDCl_3_ with TMS as internal standard. All coupling constants (*J*) are given in Hz. The symbols α and β indicate non-equivalent geminal protons. High resolution mass spectra were recorded on a Thermo Fisher Exactive electrospray mass spectrometer. The specific rotation was measured on a Perkin Elmer 341 polarimeter. TLC plates (Silica Gel 60 F254) were purchased from Merck, Germany.

Individual components were isolated using semipreparative HPLC (Turbo LC 200 chromatograph (PerkinElmer), detection using a diode matrix, UV 254 nm; analytical column 4 × 100 mm packed with Kromasil C18, 5 μm; preparative column 10 × 250 mm packed with Kromasil C18, 5 μm); gradient elution was used: eluent A was 0.1% trifluoroacetic acid in distilled water (pH 2.0), eluent B was acetonitrile, and the elution rates were 1 and 4 mL·min^−1^ for the analytical and preparative columns, respectively.

At the end of the reaction (TLC), the mixture was evaporated *in vacuo* and the residue was dissolved in acetonitrile and purified by HPLC to obtain other products.

Crystals of **4j** (C_25_H_32_N_2_O_3_, M = 408.53) are orthorhombic, space group P2_1_2_1_2_1_, at 100 K: *a* = 11.8054(6), *b* = 11.8383(6), *c* = 14.8937(8) Å, *V* = 2081.5(2) Å^3^, Z = 4 (Z′ = 1), *d*_calc_ = 1.304 g·cm^−3^, μ(MoKα) = 0.90 cm^−1^, *F*_(000)_ = 880. Intensities of 32,399 reflections were measured with a Bruker SMART APEX2 CCD diffractometer [λ(MoKα) = 0.71072Å, ω-scans, 2θ<66.4°] and 8025 independent reflections [R_int_ = 0.0363] were used in further refinement. The structure was solved by direct method and refined by the full-matrix least-squares technique against *F*^2^ in the anisotropic-isotropic approximation. The hydrogen atoms of the NH and OH groups were found in difference Fourier synthesis. The H(C) atom positions were calculated. All the hydrogen atoms with the exception of the H(2N) and H(9O) atoms were refined in the isotropic approximation within the riding model. For **4j**, the refinement converged to wR2 = 0.1070 and GOF = 0.992 for all independent reflections [R1 = 0.0409 was calculated against *F* for 7230 observed reflections with *I* > 2σ(I)]. All calculations were performed using SHELXTL PLUS 5.0 (Sheldrick, 2008). CCDC 940072 contains the supplementary crystallographic data for **4j**. These data can be obtained free of charge via http://www.ccdc.cam.ac.uk/conts/retrieving.html (or from the CCDC, 12 Union Road, Cambridge, CB21EZ, UK; or deposit@ccdc.cam.ac.uk).

Compound **1** was prepared using the method reported in Klochkov et al. (2006).

### General Procedure for the Preparing of Compounds 4, 6

A mixture of the epoxyalantolactone (**1**) (1 mmol) and the corresponding amine (1.1 mmol) was dissolved in 5 ml of methanol and stirred at room temperature. The products **5**, **8**, **9**, **16** were precipitated from the reaction mixture and recrystallized from methanol twice. In the case of other products at the end of the reaction (TLC) the mixture was evaporated *in vacuo* and the residue was dissolved in acetonitrile and purified by HPLC.

*(2aR,4aR,5aR,9S,9aR,9bR,9cS)-1-Benzyl-9a-hydroxy-5a,9-dimethyldodecahydrobenzo[g]-furo[4,3,2-cd]indol-3(1H)-one* (**4a**). Prepared from the commercially available benzylamine; reaction time 24 h; yield 70 %. White crystals, mp 211–212°C. ${\left[\alpha \right]}_{\text{D}}^{20}$ = +74° (c = 0.1; MeOH). IR spectrum, ν, cm^−1^: 1,761 (OC = O), 3,559 (OH). MS (ESI): *m/z* = 356.2228 (M+H^+^). C_22_H_30_NO_3._ Calculated, *m/z* = 356.2220. ^1^H NMR spectrum, δ, ppm: 1.26 (dt, *J* = 13.4, 2.0 Hz, 1H, 6-H_α_) 1.31 (d, *J* = 6.8 Hz, 3H, 9-Me), 1.33–1.36 (m, 1H, 8-H_α_), 1.38 (s, 3H, 5a-Me), 1.44 (dtt, *J* = 13.9, 4.1, 1.9 Hz, 1H, 7-H_α_), 1.61 (td, *J* = 13.6, 4.3 Hz, 1H, 6-H_β_), 1.69 (dd, *J* = 15.1, 1.8 Hz, 1H, 5-H_α_), 1.93 (qt, *J* = 13.7, 4.3 Hz, 1H, 7-H_β_), 2.04 (t, *J* = 6.8, 1H, 9-H), 2.06 (dt, *J* = 13.6, 4.1 Hz, 1H, 8-H_β_), 2.12 (dd, *J* = 15.0, 4.3 Hz, 1H, 5-H_β_), 2.32 (dd, *J* = 10.3, 7.3 Hz, 1H, 2-H_α_), 2.74 (d, *J* = 10.1 Hz, 1H, 9b-H), 3.04 (d, *J* = 14.7, 1H, 1-CH_α_), 3.14 (dd, *J* = 9.8, 7.1 Hz, 1H, 2a-H), 3.47 (td, *J* = 9.95, 7.3 Hz, 1H, 9c-H), 3.69 (d, *J* = 10.1 Hz, 1H, 2-H_β_), 4.55 (d, *J* = 14.7 Hz, 1H, 1-CH_β_), 4.81 (ddd, *J* = 7.1, 4.5, 2.0 Hz, 1H, 4a-H), 7.25 (dq, *J* = 8.5, 4.3 Hz, 1H, 4′-H), 7.33 (br.s., 2H, 2′-H, 6′-H), 7.34 (br.s., 2H, 3′-H, 5′-H). ^13^C NMR spectrum, δ, ppm: 15.63 (C-7), 17.09 (9-Me), 21.72 (5a-Me), 28.52 (C-8), 35.01 (C-5a), 37.21 (C-6), 39.00 (C-9), 39.72 (C-5), 41.22 (C-9c), 44.58 (C-2a), 59.78 (C-2), 64.90 (1-CH_2_), 75.78 (C-9b), 76.63 (C-9a), 77.38 (C-4a), 126.81 (C-4′), 127.31 (C-3′), 127.31 (C-5′), 128.50 (C-2′), 128.50 (C-6′), 138.98 (C-1′), 178.76 (C-3).

*(2aR,4aR,5aR,9S,9aR,9bR,9cS)-9a-Hydroxy-5a,9-dimethyl-1-phenethyldodecahydro-benzo[g]furo[4,3,2-cd]indol-3(1H)-one* (**4b**). Prepared from the commercially available phenetylamine; reaction time 12 h; yield 68 %. White crystals, mp 220–221°C. ${\left[\alpha \right]}_{\text{D}}^{20}$ = +55° (c = 0.1; MeOH). IR spectrum, ν, cm^−1^: 1,756(OC = O), 3,600 (OH). MS (ESI): *m/z* = 370.2380 (M+H^+^). C_23_H_32_NO_3._ Calculated, *m/z* = 370.2376. ^1^H NMR spectrum, δ, ppm: 1.18 (dt, *J* = 13.6, 2.3 Hz, 1H, 6-H_α_), 1.23 (s, 3H, 5a-Me), 1.27 (dt, *J* = 13.3, 1.7 Hz, 1H, 8-H_α_), 1.36 (d, *J* = 6.8 Hz, 3H, 9-Me), 1.40 (dtt, *J* = 13.6, 6.8, 2.3 Hz, 1H, 7-H_α_), 1.54 (td, *J* = 13.6, 4.5 Hz, 1H, 6-H_β_), 1.60 (dd, *J* = 15.1, 1.8 Hz, 1H, 5-H_α_), 1.89 (qt, *J* = 13.6, 4.3 Hz, 1H, 7-H_β_), 1.92–1.95 (m, 1H, 9-H), 1.99 (dt, *J* = 13.3, 4.0 Hz, 1H, 8-H_β_), 2.05 (dd, *J* = 15.1, 4.7 Hz, 1H, 5-H_β_), 2.09 (ddd, *J* = 11.6, 10.2, 6.8 Hz, 1H, 1-CH_α_), 2.49 (dd, *J* = 9.9, 7.6 Hz, 1H, 2-H_α_), 2.53 (d, *J* = 10.2 Hz, 1H, 9b-H), 2.79 (ddd, *J* = 13.3, 11.6, 5.9 Hz, 1H, PhCH_α_), 2.83 (ddd, *J* = 13.3, 11.6, 6.0 Hz, 1H, PhCH_β_), 3.21 (ddd, *J* = 9.3, 7.6, 0.8 Hz, 1H, 2a-H), 3.25 (td, *J* = 11.6, 5.7 Hz, 1H, 1-CH_β_), 3.39 (td, *J* = 10.2, 7.6 Hz, 1H, 9c-H), 3.95 (d, *J* = 9.6 Hz, 1H, 2-H_β_), 4.77 (ddd, *J* = 7.3, 4.3, 2.0 Hz, 1H, 4a-H), 7.17 (dd, *J* = 7.4, 1.4 Hz, 2H, 2′-H, 6′-H), 7.21 (tt, *J* = 7.4, 1.4, 1H, 4′-H), 7.29 (tt, *J* = 7.4, 1.4 Hz, 2H, 3′-H, 5′-H). ^13^C NMR spectrum, δ, ppm: 15.61 (C-7), 16.70 (9-Me), 21.54 (5a-Me), 28.41 (C-8), 34.94 (C-5a), 35.77 (PhCH_2_), 37.07 (C-6), 38.77 (C-9), 39.51 (C-5), 40.65 (C-9c), 44.87 (C-2a), 59.63 (C-2), 64.12 (1-CH_2_), 76.19 (C-9b), 76.22 (C-9a), 77.48 (C-4a), 126.20 (C-4′), 128.49 (C-3′), 128.49 (C-5′), 128.66 (C-2′), 128.66 (C-6′), 139.96 (C-1′), 179.03 (C-3).

*(2aR,4aR,5aR,9S,9aR,9bR,9cS)-9a-Hydroxy-5a,9-dimethyl-1-(3-phenylpropyl)-dodecahydrobenzo[g]furo[4,3,2-cd]indol-3(1H)-one* (**4c**). Prepared from the commercially available 3-phenylpropylamine; reaction time 12 h; yield 77%. White crystals, mp 174–175°C. ${\left[\alpha \right]}_{\text{D}}^{20}$ = +40° (c = 0.1; MeOH). IR spectrum, ν, cm^−1^: 1,758 (OC = O), 3,560 (OH). MS (ESI): *m/z* = 384.2540 (M+H^+^). C_24_H_34_NO_3._ Calculated, *m/z* = 384.2533. ^1^H NMR spectrum, δ, ppm: 1.09 (d, *J* = 6.8 Hz, 3H, 9-Me), 1.13–1.16 (m, 1H, 6-H_α_), 1.17 (s, 3H, 5a-Me), 1.23 (dtt, *J* = 13.3, 4.2, 2.0 Hz, 1H, 8-H_α_), 1.37 (dtt, *J* = 14.1, 4.2, 2.0 Hz, 1H, 7-H_α_), 1.52 (td, *J* = 13.6, 5.0 Hz, 1H, 6-H_β_), 1.57 (dd, *J* = 15.1, 1.7 Hz, 1H, 5-H_α_), 1.77–1.84 (m, 4H, 9-H, 1-CH_α_, 1-CH_2_CH_2_), 1.86–1.92 (m, 1H, 7-H_β_), 1.96 (dt, *J* = 13.3, 4.3 Hz, 1H, 8-H_β_), 2.02 (dd, *J* = 15.1, 4.3 Hz, 1H, 5-H_β_), 2.34 (dd, *J* = 9.9, 7.6 Hz, 1H, 2-H), 2.48 (d, *J* = 10.5, 1H, 9b-H), 2.57 (td, *J* = 13.8, 7.5 Hz, 1H, PhCH_α_), 2.59 (td, *J* = 13.8, 7.5 Hz, 1H, PhCH_β_), 2.99–3.07 (m, 1H, 1-CH_β_), 3.14 (dd, *J* = 9.9, 7.4 Hz, 1H, 2a-H), 3.34 (td, *J* = 10.2, 7.5 Hz, 1H, 9c-H), 3.80 (d, *J* = 9.8, 1H, 2-H_β_), 4.74 (ddd, *J* = 7.4, 4.3, 2.0 Hz, 1H, 4a-H), 7.16 (d, *J* = 7.3 Hz, 2H, 2′-H, 6′-H), 7.18 (t, *J* = 7.3 Hz, 1H, 4′-H) 7.27 (t, *J* = 7.3 Hz, 2H, 3′-H, 5′-H). ^13^C NMR spectrum, δ, ppm: 15.59 (C-7), 16.13 (9-Me), 21.50 (5a-Me), 28.38 (C-8), 30.45 (1-CH_2_*C*H_2_), 33.73 (PhCH_2_), 34.91 (C-5a), 37.06 (C-6), 38.82 (C-9), 39.51 (C-5), 40.62 (C-9c), 44.82 (C-2a), 59.46 (C-2), 61.75 (1-CH_2_), 76.28 (C-9b), 76.32 (C-9a), 77.40 (C-4a), 125.90 (C-4′), 128.33 (C-3′,C 5′), 128.41 (C-2′, C-6′), 141.81 (C-1′), 179.02 (C-3).

*(2aR,4aR,5aR,9S,9aR,9bR,9cS)-9a-Hydroxy-1-(4-methoxybenzyl)-5a,9-dimethyldodecahydro-benzo[g]furo[4,3,2-cd]indol-3(1H)-one* (**4d**). Prepared from the commercially available 4-methoxybenzylamine; reaction time 18 h; yield 76%. White crystals, mp 166–168°C. ${\left[\alpha \right]}_{\text{D}}^{20}$ = +45° (c = 0.1; MeOH). IR spectrum, ν, cm^−1^: 1,032 and 1,245 (MeO), 1,760 (OC = O), 3,560 (OH). MS (ESI): *m/z* = 386.2336 (M+H^+^). C_23_H_32_NO_4._ Calculated, *m/z* = 386.2331 ^1^H NMR spectrum, δ, ppm: 1.19–1.24 (m, 1H, 6-H_α_), 1.26–1.29 (m, 1H, 8-H_α_), 1.29 (d, *J* = 6.6 Hz, 3H, 9-Me), 1.34 (s, 3H, 5a-Me), 1.42 (dtt, *J* = 13.6, 6.6, 2.4 Hz, 1H, 7-H_α_), 1.58 (td, *J* = 13.5, 4.7 Hz, 1H, 6-H_β_), 1.65 (d, *J* = 15.2 Hz, 1H, 5-H_α_), 1.90 (qt, *J* = 13.6, 4.2 Hz, 1H, 7-H_β_), 1.98–2.02 (m, 1H, 9-H), 2.03 (dt, *J* = 13.6, 3.9 Hz, 1H, 8-H_β_), 2.08 (dd, *J* = 15.2, 4.3 Hz, 1H, 5-H_β_), 2.28 (dd, *J* = 10.1, 7.3 Hz, 1H, 2-H_α_), 2.68 (d, *J* = 10.1 Hz, 1H, 9b-H), 2.93 (d, *J* = 14.3 Hz, 1H, 1-CH_α_), 3.10 (dd, *J* = 9.8, 7.3 Hz, 1H, 2a-H), 3.40–3.47 (m, 1H, 9c-H), 3.62 (d, *J* = 10.5 Hz, 1H, 2-H_β_), 3.80 (s, 3H, OMe), 4.45 (d, *J* = 14.3 Hz, 1H, 1-CH_β_), 4.78 (ddd, *J* = 7.0, 4.3, 1.7 Hz, 1H, 4a-H), 6.85 (d, *J* = 8.4 Hz, 2H, 3′-H, 5′-H), 7.22 (d, *J* = 8.4 Hz, 2H, 2′-H, 6′-H). ^13^C NMR spectrum, δ, ppm: 15.63 (C-7), 17.10 (9-Me), 21.71 (5a-Me), 28.50 (C-8), 35.00 (C-5a), 37.19 (C-6), 39.00 (C-9), 39.71 (C-5), 41.25 (C-9c), 44.51 (C-2a), 55.28 (OMe), 59.71 (C-2), 64.28 (1-CH_2_), 75.81 (C-9b), 76.62 (C-9a), 77.36 (C-4a), 113.89 (C-3′), 113.89 (C-5′), 128.39 (C-2′), 128.39 (C-6′), 131.03 (C-1′), 158.49 (C-4′), 178.79 (C-3).

*(2aR,4aR,5aR,9S,9aR,9bR,9cS)-9a-Hydroxy-1-[2-(4-methoxyphenyl)ethyl]-5a,9-dimethyl-dodecahydrobenzo[g]furo[4,3,2-cd]indol-3(1H)-one* (**4e**) and *(3S,3aS,4S,4aR,5S,8aR,9aR)-4,4a-epoxy-3-[2-(4-methoxyphenyl)ethyl]-5,8a-dimethyl-decahydronaphtho[2,3-b]furan-2-one* (**6c**). Prepared from the commercially available 2-(4-methoxyphenyl)ethylamine; reaction time 12 h; yield 10 and 76%, respectively (after separation by HPLC).

#### Compound 4e

White crystals, mp 137–138°C. ${\left[\alpha \right]}_{\text{D}}^{20}$ = +46° (c = 0.1; MeOH). IR spectrum, ν, cm^−1^: 1,759 (OC = O), 3,600 (OH). MS (ESI): *m/z* = 400.2480 (M+H^+^). C_24_H_34_NO_4_ Calculated, *m/z* = 400.2482. ^1^H NMR spectrum, δ, ppm: 1.15–1.21 (m, 1H, 6-H_α_), 1.23 (s, 3H, 5a-Me), 1.24–1.27 (m, 1H, 8-H_α_), 1.35 (d, *J* = 6.8 Hz, 3H, 9-Me), 1.40 (ddd, *J* = 13.5, 4.4, 4.1 Hz, 1H, 7-H_α_), 1.54 (td, *J* = 13.4, 4.4 Hz, 1H, 6-H_β_), 1.60 (dd, *J* = 14.5, 2.4 Hz, 1H, 5-H_α_), 1.89 (dt, *J* = 13.4, 4.1 Hz, 1H, 7-H_β_), 1.91–1.95 (m, 1H, 9-H), 1.99 (dt, *J* = 13.4, 4.1 Hz, 1H, 8-H_β_), 2.02–2.10 (m, 1H, 1-CH_α_), 2.05 (dd, *J* = 14.5, 4.2 Hz, 1H, 5-H_β_), 2.47 (dd, *J* = 9.6, 7.7 Hz, 1H, 2-H_α_), 2.52 (d, *J* = 10.4 Hz, 1H, 9b-H), 2.75 (tt, *J* = 10.7, 5.7 Hz, 2H, PhCH_2_), 3.18–3.24 (m, 2H, 2a-H, 1-CH_β_), 3.38 (td, *J* = 10.1, 7.8 Hz, 1H, 9c-H), 3.79 (s, 3H, OMe), 3.93 (d, *J* = 9.8, 1H, 2-H_β_), 4.77 (ddd, *J* = 7.4, 4.9, 2.5 Hz, 1H, 4a-H), 6.83 (dt, *J* = 8.6, 2.2 Hz, 2H, 3′-H, 5′-H), 7.08 (dt, *J* = 8.6, 2.1 Hz, 2H, 2′-H, 6′-H). ^13^C NMR spectrum, δ, ppm: 15.61 (C-7), 16.71 (9-Me), 21.53 (5a-Me), 28.41 (C-8), 34.83 (PhCH_2_), 34.93 (C-5a), 37.07 (C-6), 38.78 (C-9), 39.51 (C-5), 40.63 (C-9c), 44.87 (C-2a), 55.30 (OMe), 59.63 (C-2), 64.39 (1-CH_2_), 76.20 (C-9b), 76.22 (C-9a), 77.48 (C-4a), 113.89 (C-3′), 113.89 (C-5′), 129.57 (C-2′), 129.57 (C-6′), 132.04 (C-1′), 158.04 (C-4′), 179.04 (C-3).

#### Compound 6c

White crystals, mp 100–101°C. ${\left[\alpha \right]}_{\text{D}}^{20}$ = +68° (c = 0.1; MeOH). IR spectrum, ν, cm^−1^: 1,027ℵ 1,236 (MeO), 1,750 (OC = O), 3,460 (NH). MS (ESI): *m/z* = 400.2474 (M+H^+^). C_24_H_34_NO_4_ Calculated, *m/z* = 400.2482. ^1^H NMR spectrum, δ, ppm: 1.13 (d, *J* = 8.0 Hz, 3H, 5-Me), 1.17 (s, 3H, 8a-Me), 1.33–1.41 (m, 2H, 5-H, 8-H_α_), 1.39–1.47 (m, 2H, 6-H), 1.47–1.51 (m, 1H, 7-H_α_), 1.53 (dd, *J* = 15.0, 1.9 Hz, 1H, 9-H_α_), 1.77–1.81 (m, 2H, 7-H_β_, 8-H_β_), 1.83 (dd, *J* = 15.0, 4.5 Hz, 1H, 9-H_β_), 2.64 (q, *J* = 6.1 Hz, 1H, 3-H), 2.70–2.77 (m, 2H, PhCH_2_), 2.79 (d, *J* = 1.9 Hz, 1H, 4-H), 2.82–2.93 (m, 2H, NHCH_2_), 2.96 (dd, *J* = 12.2, 6.1 Hz, 1H, 3-CH_α_), 2.98 (ddd, *J* = 9.3, 5.1, 2.2 Hz, 1H, 3a-H), 3.03 (dd, *J* = 12.5, 6.2 Hz, 1H, 3-CH_β_), 3.78–3.82 (s, 3H, OMe), 4.59 (ddd, *J* = 9.3, 4.5, 1.9 Hz, 1H, 9a-H), 6.85 (dt, *J* = 8.6, 2.2 Hz, 2H, 3′-H, 5′-H), 7.12 (dt, *J* = 8.6, 2.2 Hz, 2H, 2′-H, 6′-H). ^13^C NMR spectrum, δ, ppm: 16.47 (C-7), 18.16 (5-Me), 23.88 (8a-Me), 29.57 (C-6), 32.40 (C-8a), 35.51 (PhCH2), 37.06 (C-3a), 37.36 (C-5), 37.83 (C-8), 39.40 (C-9), 45.64 (C-3), 50.74 (3-CH_2_), 51.41 (NHCH_2_), 55.28 (OMe), 60.51 (C-4), 67.23 (C-4a), 76.25 (C-9a), 113.98 (C-3′), 113.98 (C-5′), 129.63 (C-2′), 129.63 (C-6′), 131.74 (C-1′), 158.14 (C-4′), 177.65 (C-2).

*(2aR,4aR,5aR,9S,9aR,9bR,9cS)-9a-Hydroxy-1-(3,4-dimethoxybenzyl)-5a,9-dimethyldodecahydro-benzo[g]furo[4,3,2-cd]indol-3(1H)-one* (**4f**). Prepared from the commercially available 3,4-dimethoxybenzylamine; reaction time 12 h; yield 84%. White crystals, mp 177–178°C. ${\left[\alpha \right]}_{\text{D}}^{20}$ = +64° (c = 0.1; MeOH). IR spectrum, ν, cm^−1^: 1,030 and 1,240 (MeO), 1,758 (OC = O), 3,560 (OH). MS (ESI): *m/z* = 416.2422 (M+H^+^). C_24_H_34_NO_5._ Calculated, *m/z* = 416.2431. ^1^H NMR spectrum, δ, ppm (*J*, Hz): 1.21–1.24 (m, 1H, 6-H_α_), 1.27 (d, *J* = 6.9 Hz, 3H, 9-Me), 1.29–1.32 (m, 1H, 8-H_α_), 1.34 (s, 3H, 5a-Me), 1.48–1.51 (m, 1H, 7-H_α_), 1.59 (td, *J* = 13.6, 4.9 Hz, 1H, 6-H_β_), 1.66 (dd, *J* = 15.3, 1.4 Hz, 1H, 5-H_α_), 1.84–1.88 (m, 1H, 7-H_β_), 1.97–2.01 (m, 1H, 9-H), 2.04 (dt, *J* = 12.9, 4.2 Hz, 1H, 8-H_β_), 2.09 (dd, *J* = 15.3, 4.2 Hz, 1H, 5-H_β_), 2.29 (dd, *J* = 10.3, 7.1 Hz, 1H, 2-H_α_), 2.69 (d, *J* = 10.1 Hz, 1H, 9b-H), 2.96 (d, *J* = 14.6 Hz, 1H, 1-CH_α_), 3.11 (dd, *J* = 9.7, 7.0 Hz, 1H, 2a-H), 3.43 (td, *J* = 9.8, 7.1 Hz, 1H, 9c-H), 3.69 (d, *J* = 10.4 Hz, 1H, 2-H_β_), 3.86 (s, 3H, 4′-OMe), 3.87 (s, 3H, 3′-OMe), 4.45 (d, *J* = 14.6 Hz, 1H, 1-CH_β_), 4.78 (ddd, *J* = 6.3, 4.2, 1.9 Hz, 1H, 4a-H), 6.82 (dd, *J* = 8.3, 1.7 Hz, 1H, 5′-H), 6.85 (dd, *J* = 8.3, 1.7 Hz, 1H, 6′-H), 6.87 (d, *J* = 1.7 Hz, 1H, 2′-H). ^13^C NMR spectrum, δ, ppm: 15.63 (C-7), 17.14 (9-Me), 21.65 (5a-Me), 28.52 (C-8), 35.04 (C-5a), 37.23 (C-6), 39.04 (C-9), 39.75 (C-5), 41.33 (C-9c), 44.49 (C-2a), 55.81 (4′-OMe), 55.95 (3′-OMe), 59.77 (C-2), 63.99 (1-CH_2_), 75.46 (C-9b), 76.69 (C-9a), 77.35 (C-4a), 110.42 (C-5′), 111.29 (C-2′), 119.13 (C-6′), 131.61 (C-1′), 147.76 (C-4′), 149.01 (C-3′), 178.72 (C-3).

*(2aR,4aR,5aR,9S,9aR,9bR,9cS)-9a-Hydroxy-1-[2-(3,4-dimethoxyphenyl)ethyl]-5a,9-dimethyldodecahydrobenzo[g]furo[4,3,2-cd]indol-3(1H)-one* (**4g**). Prepared from the commercially available 2-(3,4-dimethoxyphenyl)ethylamine; reaction time 12 h; yield 80%. White crystals, mp 185–187°C. ${\left[\alpha \right]}_{\text{D}}^{20}$ = +35° (c = 0.1; MeOH). IR spectrum, ν, cm^−1^: 1,030 and 1,240 (MeO), 1,759 (OC = O), 3,562 (OH). MS (ESI): *m/z* = 430.2596 (M+H^+^). C_25_H_36_NO_5_ Calculated, *m/z* = 430.2588. ^1^H NMR spectrum, δ, ppm: 1.18–1.21 (m, 1H, H-6α), 1.22 (s, 3H, 5a-Me), 1.27–1.30 (m, 1H, 8-H_α_), 1.32 (d, *J* = 6.6 Hz, 3H, 9-Me), 1.40 (dtt, *J* = 13.8, 6.3, 2.6 Hz, 1H, 7-H_α_), 1.54 (td, *J* = 13.8, 4.6 Hz, 1H, 6-H_β_), 1.61 (dd, *J* = 15.1, 1.6 Hz, 1H, 5-H_α_), 1.88 (qt, *J* = 13.8, 4.3 Hz, 1H, 7-H_β_), 1.92–1.95 (m, 1H, 9-H), 1.99 (dt, *J* = 13.6, 4.3 Hz, 1H, 8-H_β_), 2.05 (dd, *J* = 15.1, 4.3 Hz, 1H, 5-H_β_), 2.08 (ddd, *J* = 11.8, 9.2, 7.5 Hz, 1H, 1-CH_α_), 2.48 (dd, *J* = 9.8, 7.8 Hz, 1H, 2-H_α_), 2.52 (d, *J* = 10.4 Hz, 1H, 9b-H), 2.76 (ddd, *J* = 9.8, 6.3, 2.9 Hz, 2H, PhCH_2_), 3.21 (dd, *J* = 10.1, 7.8 Hz, 1H, 2a-H), 3.23 (td, *J* = 11.5, 6.3 Hz, 1H, 1-CH_β_), 3.39 (td, *J* = 10.1, 7.5 Hz, 1H, 9c-H), 3.86 (s, 3H, 4′-OMe), 3.88 (s, 3H, 3′-OMe), 3.95 (d, *J* = 9.8 Hz, 1H, 2-H_β_), 4.78 (ddd, *J* = 7.5, 4.3, 1.7 Hz, 1H, 4a-H), 6.69 (s, 1H, 2′-H), 6.70 (dd, *J* = 7.8, 2.0 Hz, 1H, 6′-H), 6.79 (d, *J* = 7.7 Hz, 1H, 5′-H). ^13^C NMR spectrum, δ, ppm: 15.59 (C-7), 16.78 (9-Me), 21.53 (5a-Me), 28.40 (C-8), 34.93 (C-5a), 35.33 (PhCH_2_), 37.07 (C-6), 38.74 (C-9), 39.52 (C-5), 40.68 (C-9c), 44.84 (C-2a), 55.88 (4′-OMe), 55.95 (3′-OMe), 59.62 (C-2), 64.16 (1-CH2), 76.22 (C-9b), 76.27 (C-9a), 77.45 (C-4a), 111.27 (C-5′), 112.09 (C-2′), 120.48 (C-6′), 132.58 (C-1′), 147.46 (C-4′), 148.85 (C-3′), 178.99 (C-3).

*(2aR,4aR,5aR,9S,9aR,9bR,9cS)-9a-Hydroxy-1-(2-dimethylaminoethyl)-5a,9-dimethyldodecahydro-benzo[g]furo[4,3,2-cd]indol-3(1H)-one* (**4h**). Prepared from the commercially available 2-dimethylaminoethylamine; reaction time 24 h; yield 83%. White crystals, mp 174–176°C. ${\left[\alpha \right]}_{\text{D}}^{20}$ = +28° (c = 0.1; MeOH). IR spectrum, ν, cm^−1^: 1,760 (OC = O), 3,560 (OH). MS (ESI): *m/z* = 337.2478 (M+H^+^). C_19_H_33_N_2_O_3_ Calculated, *m/z* = 337.2486. ^1^H NMR spectrum, δ, ppm: 1.13–1.17 (m, 1H, 6-H_α_), 1.19 (s, 3H, 5a-Me), 1.27–1.29 (m, 1H, 8-H_α_), 1.30 (d, *J* = 7.0 Hz, 3H, 9-Me), 1.37–1.45 (m, 1H, 7-H_α_), 1.54 (dd, *J* = 13.5, 5.2 Hz, 1H, 6-H_β_), 1.59 (dd, *J* = 15.0, 1.8 Hz, 1H, 5-H_α_), 1.88 (qt, *J* = 13.7, 4.4 Hz, 1H, 7-H_β_), 1.94–2.11 (m, 4H, 8-H_β_, 5-H_β_, 9-H, 1-CH_α_), 2.24 (s, 6H, NMe_2_), 2.42 (td, *J* = 10.9, 5.2 Hz, 1 H, CH_α_NMe_2_), 2.42 (dd, *J* = 9.8, 7.8 Hz, 1H, 2-H_α_), 2.48 (td, *J* = 11.7, 5.2 Hz, 1H, CH_β_NMe_2_), 2.56 (d, *J* = 10.4, 1H, 9b-H), 3.17 (dd, *J* = 9.8, 7.0 Hz, 1H, 2a-H), 3.22 (td, *J* = 11.7, 5.2 Hz, 1H, 1-CH_β_), 3.36 (td, *J* = 10.1, 7.5 Hz, 1H, 9c-H), 3.78 (d, *J* = 9.8 Hz, 1H, 2-H_β_), 4.76 (ddd, *J* = 7.1, 4.6, 2.1 Hz, 1H, 4a-H). ^13^C NMR spectrum, δ, ppm: 15.59 (C-7), 16.60 (9-Me), 21.54 (5a-Me), 28.43 (C-8), 34.92 (C-5a), 37.08 (C-6), 38.88 (C-9), 39.51 (C-5), 40.43 (C-9c), 44.93 (C-2a), 46.11 (NMe_2_), 58.28 (CH_2_NMe_2_), 59.83 (C-2), 60.41 (1-CH_2_), 76.24 (C-9a), 76.44 (C-9b), 77.42 (C-4a), 178.93 (C-3).

*(2aR,4aR,5aR,9S,9aR,9bR,9cS)-9a-Hydroxy-5a,9-dimethyl-1-(2-morpholin-4-ylethyl)dodecahydrobenzo[g]furo[4,3,2-cd]indol-3(1H)-one* (**4i**). Prepared from the commercially available 2-morpholin-4-ylethylamine; reaction time 12 h; yield 70%. White crystals, mp 192–194°C. ${\left[\alpha \right]}_{\text{D}}^{20}$ = +18° (c = 0.1; MeOH). IR spectrum, ν, cm^−1^: 1,760 (OC = O), 3,566 (OH). MS (ESI): *m/z* = 379.2599 (M+H^+^). C_21_H_35_N_2_O_4._ Calculated, *m/z* = 379.2591. ^1^H NMR spectrum, δ, ppm: 1.21 (s, 3H, 5a-Me), 1.22–1.24 (m, 1H, 6-H_α_), 1.30–1.32 (m, 1H, 8-H_α_), 1.33 (d, *J* = 7.0 Hz, 3H, 9-Me), 1.39–1.49 (m, 1H, 7-H_α_), 1.56 (td, *J* = 13.5, 5.4 Hz, 1H, 6-H_β_), 1.62 (dd, *J* = 15.1, 1.6 Hz, 1H, 5-H_α_), 1.90 (dt, *J* = 13.8, 4.3 Hz, 1H, 7-H_β_), 1.97–2.03 (m, 1H, 8-H_β_), 2.01–2.05 (m, 1H, 9-H), 2.03–2.08 (m, 1H, 1-CH_α_), 2.07 (ddd, *J* = 15.1, 4.3, 0.5 Hz, 1H, 5-H_β_), 2.45 (ddd, *J* = 9.7, 8.1, 0.5 Hz, 1H, 2-H_α_), 2.47–2.56 (m, 6H, 6′-H, 2′-H, 1-CH_2_CH_2_), 2.59 (d, *J* = 10.5 Hz, 1H, 9b-H), 3.20 (dd, *J* = 10.1, 7.4 Hz, 1H, 2a-H), 3.29 (td, *J* = 12.4, 6.3 Hz, 1H, 1-CH_β_), 3.40 (td, *J* = 10.1, 7.4 Hz, 1H, 9c-H), 3.66–3.80 (m, 4H, 5′-H, 3′-H), 3.83 (d, *J* = 9.7 Hz, 1H, 2-H_β_), 4.79 (ddd, *J* = 7.1, 4. 8, 1.9 Hz, 1H, 4a-H). ^13^C NMR spectrum, δ, ppm: 15.56 (C-7), 16.72 (9-Me), 21.53 (5a-Me), 28.42 (C-8), 34.92 (C-5a), 37.06 (C-6), 38.84 (C-9), 39.49 (C-5), 40.44 (C-9c), 44.90 (C-2a), 54.19 (C-2′), 54.19 (C-6′), 57.62 (1-CH_2_*C*H_2_), 59.11 (1-CH_2_), 59.82 (C-2), 66.83 (C-3′), 66.83 (C-5′), 76.26 (C-9a), 76.39 (C-9b), 77.38 (C-4a), 178.86 (C-3).

*(2aR,4aR,5aR,9S,9aR,9bR,9cS)-9a-Hydroxy-1-[2-(1H-indol-3-yl)ethyl]-5a,9-dimethyl-dodecahydrobenzo[g]furo[4,3,2-cd]indol-3(1H)-one* (**4j**). Prepared from the commercially available tryptamine; reaction time 24 h; yield 54%. White crystals, mp 235–236°C (MeOH). ${\left[\alpha \right]}_{\text{D}}^{20}$ = +32° (c = 0.1; MeOH). IR spectrum, ν, cm^−1^: 1,770 (C = O), 3,420 (NH), 3,570 (OH). Found, *m/z*: 409.2496 [M+H]^+^. C_25_H_33_N_2_O_3_. Calculated, *m/z*: 409.2491. H NMR spectrum, δ, ppm: 1.16–1.22 (m, 2H, 6-H_α_,8-H_α_), 1.25 (s, 3H, 5a-Me), 1.34 (d, *J* = 6.8 Hz, 3H, 9-Me), 1.36–1.44 (m, 1H, 7-H_α_), 1.54 (td, *J* = 13.4, 4.1 Hz, 1H, 7-H_β_), 1.61 (d, *J* = 15.0 Hz, 1H, 5-H_α_), 1.82–1.94 (m, 2H, 6-H_β_, 8-H_β_), 1.94–2.01 (m, 1H, 9-H), 2.05 (dd, *J* = 15.0, 4.3 Hz, 1H, 5-H_β_), 2.24 (td, *J* = 11.7, 5.2 Hz, 1H, 1-CH_α_), 2.56 (dd, *J* = 9.8, 7.4 Hz, 1H, 2-H_α_), 2.57 (d, *J* = 10.2 Hz, 1H, 9b-H); 2.97 (ddd, *J* = 14.2, 11.7, 5.2 Hz, 1H, CH_α_Ind), 3.04 (ddd, *J* = 14.2, 11.7, 5.2 Hz, 1H, CH_β_Ind); 3.20–3.26 (m, 1H, 2a-H), 3.31 (td, *J* = 11.8, 5.2 Hz, 1H, 1-CH_β_), 3.36–3.45 (m, 1H, 9c-H), 4.03 (d, *J* = 9.8 Hz, 1H, 2-H_β_), 4.76–4.82 (m, 1H, 4a-H), 6.99 (d, *J* = 2.0 Hz, 1H, 2′-H), 7.13 (ddd, *J* = 7.9, 7.0, 0.9 Hz, 1H, 6′-H), 7.20 (ddd, *J* = 7.9, 7.0, 1.2 Hz, 1H, 5′-H), 7.37 (dt, *J* = 7.9, 1.2 Hz, 1H, 7′-H), 7.59 (dd, *J* = 7.9, 0.9 Hz, 1H, 4′-H); 7.98 (br. s., 1H, NH). ^13^C NMR spectrum, δ, ppm: 15.6 (C-7), 16.5 (9-Me), 21.7 (5a-Me), 24.9 (CH_2_Ind), 28.4 (C-8), 35.0 (C-5a), 37.1 (C-6), 38.9 (C-9), 39.6 (C-5), 40.7 (C-9c), 44.9 (C-2a), 56.3 (C-2), 63.0 (1-CH_2_); 75.5 (C-9a), 76.3 (C-9b), 77.3 (C-4a), 111.1 (C-7′), 114.3 (C-3′), 118.9 (C-4′), 119.3 (C-6′), 121.3 (C-2′), 122.1 (C-5′),127.7 (C-3a′), 136.2 (C-7a′, 179.1 (C-3).

*(2aR,4aR,5aR,9S,9aR,9bR,9cS)-9a-Hydroxy-1-[2-(5-methoxy-1H-indol-3-yl)ethyl]-5a,9-dimethyl-dodecahydrobenzo[g]furo[4,3,2-cd]indol-3(1H)-one* **(4k)**. Prepared from the commercially available 5-methoxytryptamine; reaction time 24 h; yield 76%. White crystals, mp 240–241°C (MeOH). ${\left[\alpha \right]}_{\text{D}}^{20}$ = +16° (c = 0.1; MeOH). IR spectrum, ν cm^−1^: 1,762 (C = O), 3,420 (NH), 3,570 (OH). Found, *m/z*: 439.2583 [M+H]^+^. C_26_H_35_N_2_O_4_. Calculated, *m/z*: 439.2591. ^1^H NMR spectrum, δ, ppm: 1.15–1.24 (m, 2H, 6-H_α_, 8-H_α_), 1.25 (s, 3H, 5a-Me), 1.35 (d, *J* = 6.7 Hz, 3H, 9-Me), 1.37–1.42 (m, 1H, 7-H_α_), 1.54 (td, *J* = 13.7, 4.6, 1H, 7-H_β_), 1.61 (dd, *J* = 14.9, 2.0 Hz, 1H, 5-H_α_), 1.85–1.90 (m, 1H, 8-H_β_), 1.91–1.94 (m, 1H, 9-H), 1.95–2.00 (m, 1H, 6-H_β_), 2.05 (dd, *J* = 15.1, 4.4 Hz, 1H, 5-H_β_), 2.21 (td, *J* = 11.6, 5.5 Hz, 1H, 1-CH_α_), 2.54 (dd, *J* = 9.8, 7.4 Hz, 1H, 2-H_α_), 2.55 (d, *J* = 10.2 Hz, 1H, 9b-H), 2.91 (ddd, *J* = 14.3, 11.6, 5.2 Hz, 1H, CH_α_Ind), 2.96 (ddd, *J* = 14.3, 11.6, 5.2 Hz, 1H, CH_β_Ind), 3.23 (dd, *J* = 9.9, 7.5 Hz, 1H, 2a-H); 3.31 (td, *J* = 11.6, 5.5 Hz, 1H, 1-CH_β_), 3.39 (td, *J* = 10.2, 7.5 Hz, 1H, 9c-H), 3.88 (s, 3H, OMe), 4.03 (d, *J* = 9.8 Hz, 1H, 2-H_β_), 4.79 (ddd, *J* = 7.1, 4.7, 2.0 Hz, 1H, 4a-H), 6.86 (dd, *J* = 8.9, 2.4 Hz, 1H, 6′-H), 6.95 (d, *J* = 2.1 Hz, 1H, 2′-H), 7.02 (d, *J* = 2.4 Hz, 1H, 4′-H), 7.25 (d, *J* = 8.9 Hz, 1H, 7′-H), 7.90 (br. s., 1H, NH). ^13^C NMR spectrum, δ, ppm: 15.6 (C-7), 16.6 (9-Me), 21.6 (5a-Me), 24.9 (CH_2_Ind), 28.4 (C-8), 35.0 (C-5a), 37.1 (C-6), 38.9 (C-9), 39.5 (C-5), 40.7 (C-9c), 44.9 (C-2a), 56.0 (OMe), 59.7 (C-2), 62.8 (1-CH_2_), 76.3 (C-9b), 77.3 (C-9a), 77.5 (C-4a), 100.7 (C-4′), 111.9 (C-7′), 112.3 (C-6′), 114.1 (C-3′), 122.1 (C-2′), 127.8 (C-3a′), 131.3 (C-7a′),154.0 (C-5′), 179.1 (C-3).

*(3S,3aS,4S,4aR,5S,8aR,9aR)-4,4a-Epoxy-3-[4-(4-fluorophenyl)-3,6-dihydro-2H-pyridin-1-ylme-thyl]-5,8a-dimethyldecahydronaphtho[2,3-b]furan-2-one* (**6a**). Prepared from the commercially available 4-(4-fluorophenyl)-3,6-dihydro-2*H*-pyridine; reaction time 12 h; yield 87%. White crystals, mp 157–159°C. ${\left[\alpha \right]}_{\text{D}}^{20}$ = +85° (c = 0.1; MeOH). IR spectrum, ν, cm^−1^: 1,230 (CF), 1757 (OC = O). MS (ESI): *m/z* = 426.2344 (M+H^+^). C_26_H_33_FNO_3._ Calculated, *m/z* = 426.2439. ^1^H NMR spectrum, δ, ppm: 1.18 (d, *J* = 7.6 Hz, 3H, 5-Me), 1.25 (s, 3H, 8a-Me), 1.37–1.44 (m, 1H, 5-H), 1.44–1.49 (m, 2H, 7-H), 1.49–1.54 (m, 1H, 8-H_α_), 1.52–1.58 (m, 1H, 6-H_α_), 1.64 (dd, *J* = 15.3, 2.6 Hz, 1H, 9-H_α_), 1.81–1.87 (m, 1H, 8-H_β_), 1.88 (dd, *J* = 15.3, 2.9 Hz, 1H, 9-H_β_), 1.87–1.95 (m, 1H, 6-H_β_), 2.50–2.65 (m, 2H, 5′-H), 2.70 (dt, *J* = 11.2, 5.3 Hz, 1H, 6′-H_α_), 2.87–2.93 (m, 1H, 6′-H_β_), 2.93 (dd, *J* = 13.0, 11.2 Hz, 1H, 3-CH_α_), 2.99 (dd, *J* = 13.1, 4.0 Hz, 1H, 3-CH_β_), 3.15 (dd, *J* = 16.7, 3.2 Hz, 1H, 2′-H_α_), 3.26 (dd, *J* = 11.2, 7.3 Hz, 1H, 3-H), 3.30 (dd, *J* = 11.2, 3.2 Hz, 1H, H-3a), 3.37 (dd, *J* = 16.7, 3.3 Hz, 1H, 2′-H_β_), 3.41 (br.s., 1H, 4-H), 4.66 (dt, *J* = 7.3, 2.9 Hz, 1H, 9a-H), 6.03 (t, *J* = 3.2 Hz, 1H, 3′-H), 7.05 (tt, *J* = 8.8, 2.0 Hz, 2H, 3″ -H, 5″-H), 7.38 (dd, *J* = 8.8, 5.3 Hz, 2H, 2″ -H, 6″-H). ^13^C NMR spectrum, δ, ppm: 16.56 (C-7), 17.85 (C-5-Me), 24.20 (C-8a-Me), 28.21 (C-5′), 29.66 (C-6), 32.15 (C-8a), 36.02 (C-3), 37.80 (C-8), 37.92 (C-5), 38.90 (C-9), 40.06 (C-3a), 50.08 (C-6′), 52.86 (C-2′), 55.04 (3-CH), 57.31 (C-4), 68.21 (C-4a), 76.05 (C-9a), 115.08 (C-3″), 115.26 (C-5″), 121.04 (C-3′), 126.40 (C-2″), 126.47 (C-6″), 134.27 (C-4′), 136.68 (C-1″), 162.12 (d, *J* = 246.1 Hz, C-4″), 177.04 (C-2).

*(3S,3aS,4S,4aR,5S,8aR,9aR)-4,4a-Epoxy-3-[4-(4-fluorophenyl)piperazin-1-ylmethyl]-5,8a-dimethyldecahydronaphtho[2,3-b]furan-2-one* (**6b**). Prepared from the commercially available 4-(4-fluorophenyl)piperazine; reaction time 12 h; yield 80%. White crystals, mp 180–183°C. ${\left[\alpha \right]}_{\text{D}}^{20}$ = +90° (c = 0.1; MeOH). IR spectrum, ν, cm^−1^: 1,230 (CF), 1,754 (OC = O). MS (ESI): *m/z* = 429.2560 (M+H^+^). C_25_H_34_FN_2_O_3._ Calculated, *m/z* = 429.2548. ^1^H NMR spectrum, δ, ppm: 1.18 (d, *J* = 7.9 Hz, 3 H, 5-Me), 1.24 (s, 3H, 8a-Me), 1.39–1.50 (m, 3H, 8-H, 5-H), 1.51–1.57 (m, 2H, 6-H_α_, 7-H_α_), 1.64 (dd, *J* = 14.9, 2.7 Hz, 1H, 9-H_α_), 1.88 (dt, *J* = 13.6, 4.2 Hz, 1H, 7-H_β_), 1.88 (dd, *J* = 14.9, 2.8 Hz, 1H, 9-H_β_), 1.89 (dt, *J* = 13.4, 4.2 Hz, 1H, 6-H_β_), 2.58 (ddd, *J* = 10.3, 6.5, 3.2 Hz, 1H, 2′-H_ax_), 2.58 (ddd, *J* = 10.3, 6.2, 3.4 Hz, 1H, 6′-H_ax_), 2.80–2.85 (m, 2H, 2′-H_eq_, 3′-H_eq_), 2.84 (dd, *J* = 13.2, 10.6 Hz, 1H, 3-CH_α_), 2.93 (dd, *J* = 13.2, 3.9 Hz, 1H, 3-CH_β_), 3.16 (ddd, *J* = 9.6, 6.5, 3.4 Hz, 4H, 5′-H, 3′-H), 3.24 (dd, *J* = 6.5, 2.5 Hz, 1H, 3a-H), 3.28 (dd, *J* = 10.6, 3.8, 1H, 3-H), 3.38 (s, 1H, 4-H), 4.65 (dt, *J* = 7.1, 2.8 Hz, 1H, 9a-H), 6.91 (dt, *J* = 9.1, 2.3 Hz, 1H, 3″ -H), 6.91 (dt, *J* = 9.1, 2.3, 1H, 5″-H), 6.99 (dt, *J* = 9.1, 2.3 Hz, 1H, 2″ -H) 7.01 (dt, *J* = 9.1, 2.3 Hz, 1H, 6″ -H). ^13^C NMR spectrum, δ, ppm: 16.55 (C-7), 17.85 (5-Me), 24.18 (8a-Me), 29.67 (C-6), 32.14 (C-8a), 36.02 (C-3), 37.80 (C-8), 37.93 (C-5), 38.87 (C-9), 39.76 (C-3a), 50.24 (C-3′, C-5′), 52.96 (C-2′, C-6′), 55.22 (3-CH_2_), 57.28 (C-4), 68.22 (C-4a), 76.00 (C-9a), 115.49 (C-3″), 115.67 (C-5″), 117.98 (C-2″), 118.04 (C-6″), 147.84 (C-1″), 157.32 (d, *J* = 239.40, C-4″), 176.85 (C-2).

*(3S,3aS,4S,4aR,5S,8aR,9aR)-4,4a-Epoxy-3-[2-(1H-indol-3-yl)ethyl]-5,8a-dimethyldecahydro-naphtho[2,3-b]furan-2-one* (**6d**). Prepared from the commercially available tryptamine; reaction time 0.5 h; yield 70%. White crystals, mp 183–184°C. ${\left[\alpha \right]}_{\text{D}}^{20}$ = +29° (c = 0.1; MeOH). IR spectrum, ν, cm^−1^: 1,757 (OC = O), 3,480 (NH). MS (ESI): *m/z* = 409.2505 (M+H^+^). C_25_H_33_N_2_O_3_ Calculated, *m/z* = 409.2491. ^1^H NMR spectrum, δ, ppm (*J*, Hz): 1.12 (d, *J* = 7.7 Hz, 3H, 5-Me), 1.22 (s, 3H, 8a-Me), 1.31 (m, 1H, 5-H), 1.44 (td, *J* = 10.8, 4.2 Hz, 2H, 8-H), 1.51 (dt, *J* = 13.8, 5.5 Hz, 1H, 7-H_α_), 1.51–1.55 (m, 1H, 6-H_α_), 1.61 (dd, *J* = 14.8, 2.9 Hz, 1H, 9-H_α_), 1.82 (dd, *J* = 14.8, 2.9 Hz, 1H, 9-H_β_), 1.82–1.86 (m, 1H, 6-H_β_), 1.86 (dt, *J* = 13.8, 5.5 Hz, 1H, 7-H_β_), 2.99 (br. s., 1H, 4-H), 3.02 (t, *J* = 4.5 Hz, 4H, NCH_2_CH_2_), 3.06 (ddd, *J* = 10.0, 7.4, 5.5 Hz, 1H, 3-CH_α_), 3.10 (dd, *J* = 10.0, 5.5 Hz, 1H, 3-H), 3.12 (d, *J* = 6.4, 5.5, 1.9 Hz, 1H, 3a-H), 3.14 (dd, *J* = 10.0, 5.5 Hz, 1H, 3-CH_β_), 4.60 (dt, *J* = 6.4, 2.9 Hz, 1H, 9a-H), 7.09 (d, *J* = 2.2 Hz, 1H, 2′-H), 7.15 (ddd, *J* = 8.1, 7.1, 1.3 Hz, 1H, 5′-H), 7.23 (ddd, *J* = 8.1, 7.1, 1.3 Hz, 1H, 6′-H), 7.40 (dt, *J* = 8.1, 1.3 Hz, 1H, 7′-H), 7.65 (dd, *J* = 8.1, 0.9 Hz, 1H, 4′-H), 8.11 (br. s., 1H, 1′-H). ^13^C NMR spectrum, δ, ppm: 16.57 (5-Me), 17.66 (C-7), 24.06 (8a-Me), 25.64 (CH_2_Ind), 29.65 (C-6), 32.05 (C-8a), 36.07 (C-3a), 37.82 (C-8), 37.95 (C-5), 38.84 (C-9), 42.60 (C-3), 46.32 (3-CH_2_), 50.12 (NHCH_2_), 57.66 (C-4), 68.38 (C-4a), 76.04 (C-9a), 111.23 (C-7′), 113.82 (C-3′), 118.81 (C-4′), 119.33 (C-6′), 121.91 (C-2′), 122.09 (C-5′), 127.41 (C-3a′), 136.42 (C-7a′), 177.46 (C-2).

## Conclusion

We showed that the intramolecular nucleophilic substitution in the NH-amino derivatives of natural epoxyalantolactone afforded compounds of the new heterocyclic system: hydrogenated benzo[g]furo[4,3,2-cd]indol-3(1H)-ones. For the first time we present the representatives of this kind of synthetized compounds and their molecular structures that were determined by spectral methods. Biological activity of these derivatives will be tested elsewhere for the detail see Supplementary Materials.

## Data Availability Statement

The datasets generated for this study are available on request to the corresponding author.

## Author Contributions

SK, SP, SA, MN, IA, and GA discussed and designed the experiments, data acquisition, organization of the database, and statistical analysis. SK, SP, SA, MN, IA, MA-R, VT, and GA contributed to the final design of the study, conception of the study, wrote section of the manuscript, and prepared the final version of the manuscript. All authors contributed to manuscript revision, read, and approved the submitted version.

### Conflict of Interest

GA was employed by GALLY International Biomedical Research Consulting LLC, San Antonio, Texas, USA. The remaining authors declare that the research was conducted in the absence of any commercial or financial relationships that could be construed as a potential conflict of interest.

## Footnotes

^1^According to the IUPAC rules, the enumeration of the carbon atoms in heterocyclic systems 4 and 6 is different.
