# Supplementary material for: A Novel Heterocyclic System Based on Natural Epoxyalantolactone
Source: Front Chem. 2019 Oct 1;7:655. doi: 10.3389/fchem.2019.00655 (PMC6779722; doi:10.3389/fchem.2019.00655)
Supplement: Supplementary file 1 [file Data_Sheet_1.pdf]

NMR spectra for compounds 4a-k and compounds 6a-d.

^{1}H and ^{13}C NMR spectra were obtained on a Bruker AVANCE III instrument (500.13 and 125.78 MHz); in the interpretation of the spectrum, the symbols “α” and “β” denote nonequivalent protons at one carbon atom.

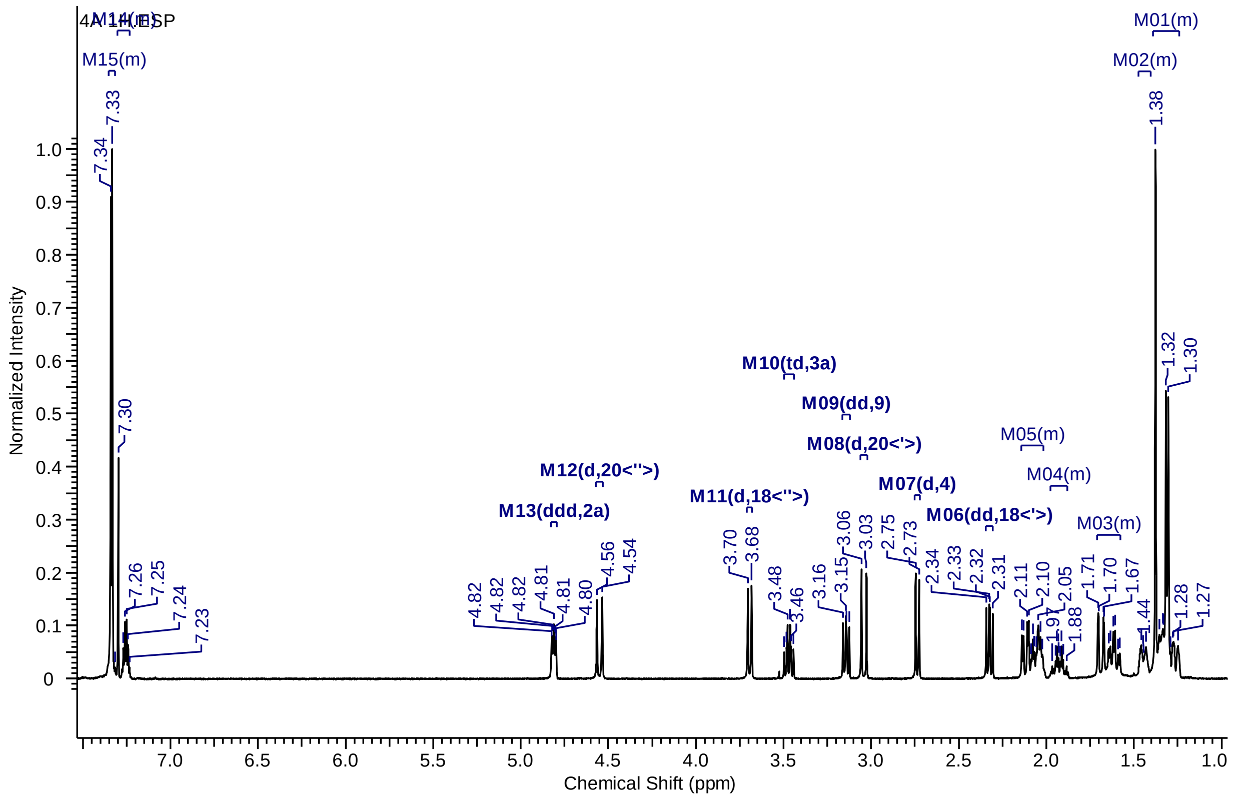
M01(m)
M02(m)
M03(m)
M04(m)
M05(m)
M06(dd,18<'>)
M07(d,4)
M08(d,20<'>)
M09(dd,9)
M10(td,3a)
M11(d,18<''>)
M12(d,20<''>)
M13(ddd,2a)
M14(m)
M15(m)
Normalized Intensity
Chemical Shift (ppm)

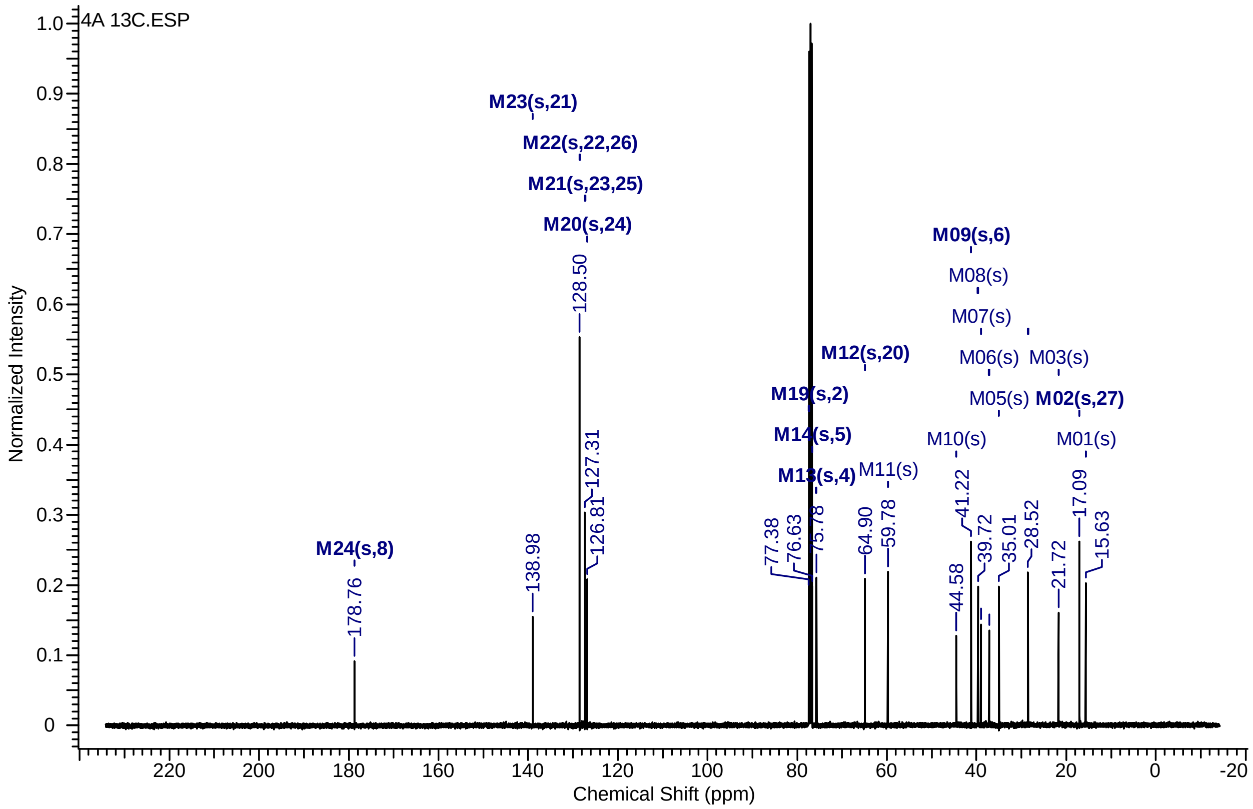
4A 13C.ESP
Normalized Intensity
Chemical Shift (ppm)
M24(s,8)
M23(s,21)
M22(s,22,26)
M21(s,23,25)
M20(s,24)
M19(s,2)
M14(s,5)
M13(s,4)
M12(s,20)
M11(s)
M10(s)
M09(s,6)
M08(s)
M07(s)
M06(s)
M05(s)
M03(s)
M02(s,27)
M01(s)
178.76
138.98
128.50
127.31
126.81
77.38
76.63
75.78
64.90
59.78
44.58
41.22
39.72
35.01
28.52
21.72
17.09
15.63

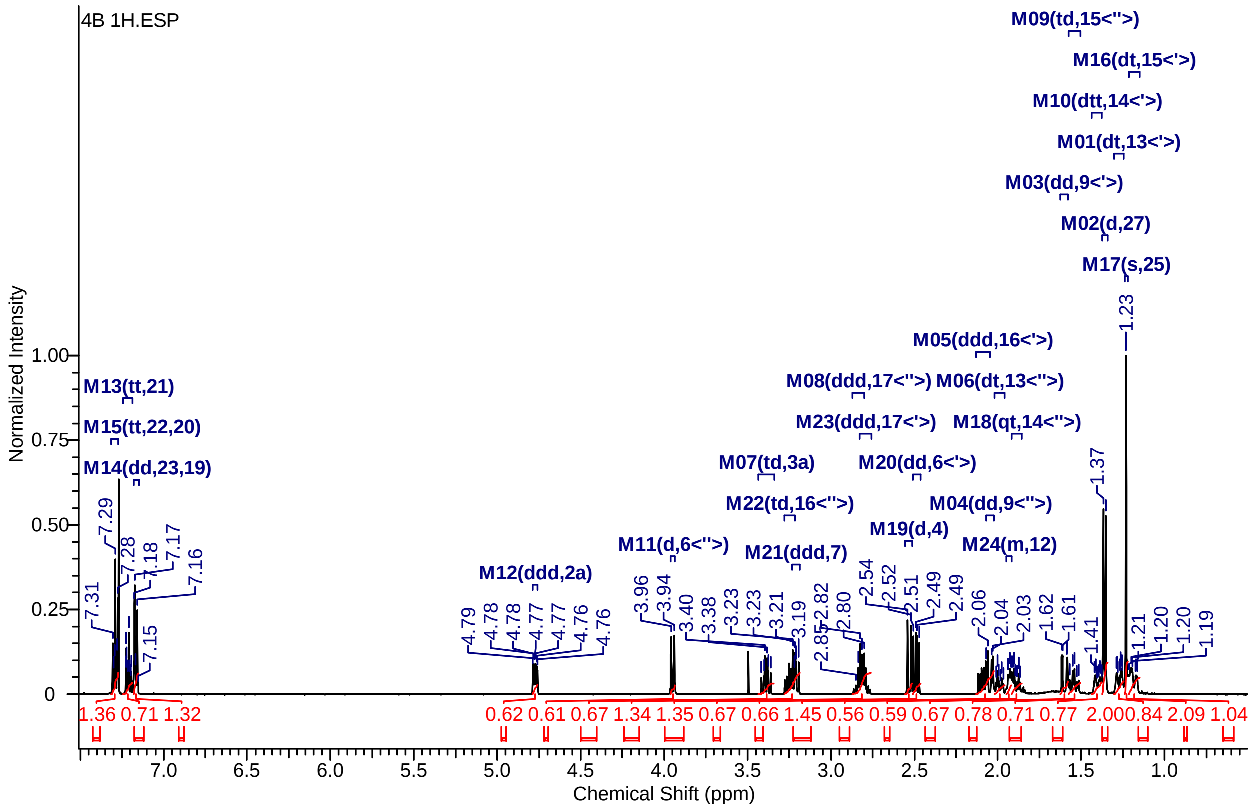
4B 1H.ESP
Normalized Intensity
Chemical Shift (ppm)
M09(td,15<">)
M16(dt,15<'>)
M10(dtt,14<'>)
M01(dt,13<'>)
M03(dd,9<'>)
M02(d,27)
M17(s,25)
M05(ddd,16<'>)
M08(ddd,17<">)
M06(dt,13<">)
M23(ddd,17<'>)
M18(qt,14<">)
M07(td,3a)
M20(dd,6<'>)
M22(td,16<">)
M04(dd,9<">)
M19(d,4)
M11(d,6<">)
M21(ddd,7)
M24(m,12)
M12(ddd,2a)
M13(tt,21)
M15(tt,22,20)
M14(dd,23,19)
1.36 0.71 1.32 0.62 0.61 0.67 1.34 1.35 0.67 0.66 1.45 0.56 0.59 0.67 0.78 0.71 0.77 2.00 0.84 2.09 1.04

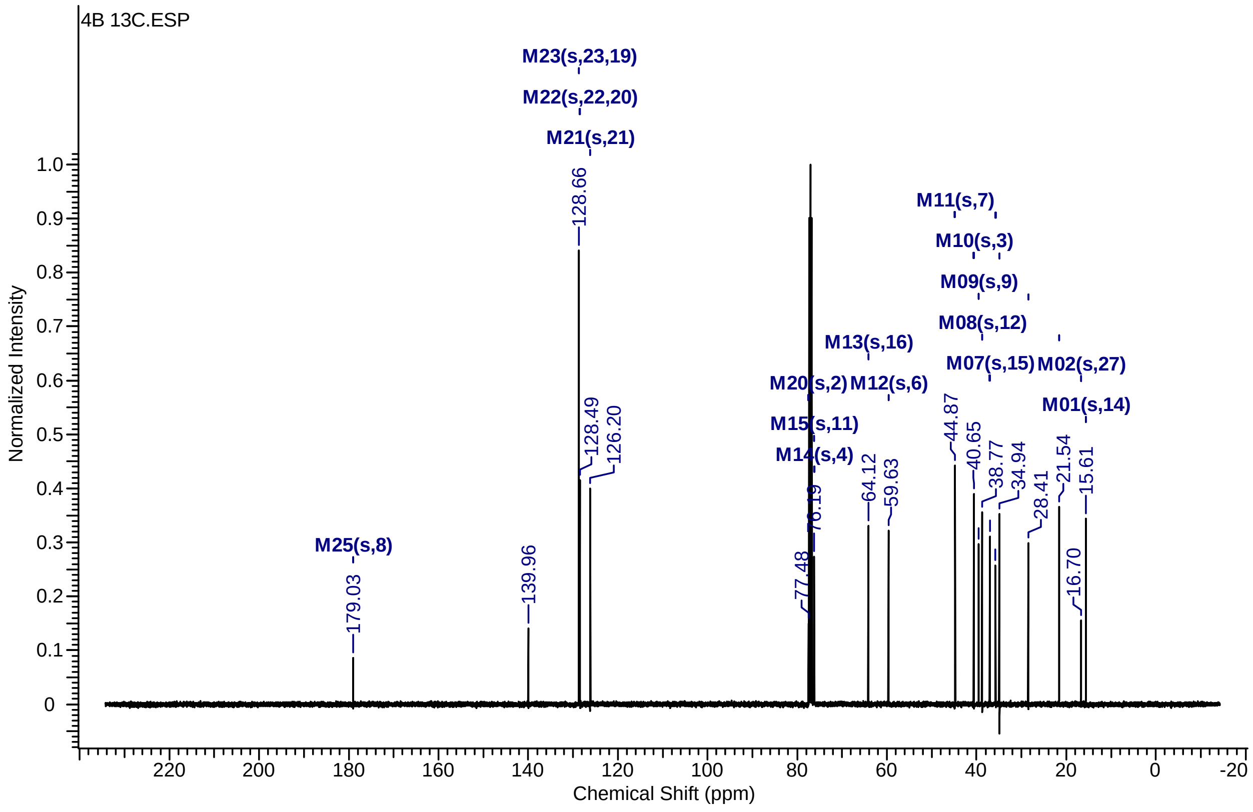
4B 13C.ESP
M23(s,23,19)
M22(s,22,20)
M21(s,21)
M25(s,8)
M20(s,2)
M15(s,11)
M14(s,4)
M13(s,16)
M12(s,6)
M11(s,7)
M10(s,3)
M09(s,9)
M08(s,12)
M07(s,15)
M02(s,27)
M01(s,14)
179.03
139.96
128.66
128.49
126.20
77.48
76.19
64.12
59.63
44.87
40.65
38.77
34.94
28.41
21.54
16.70
15.61
Normalized Intensity
Chemical Shift (ppm)

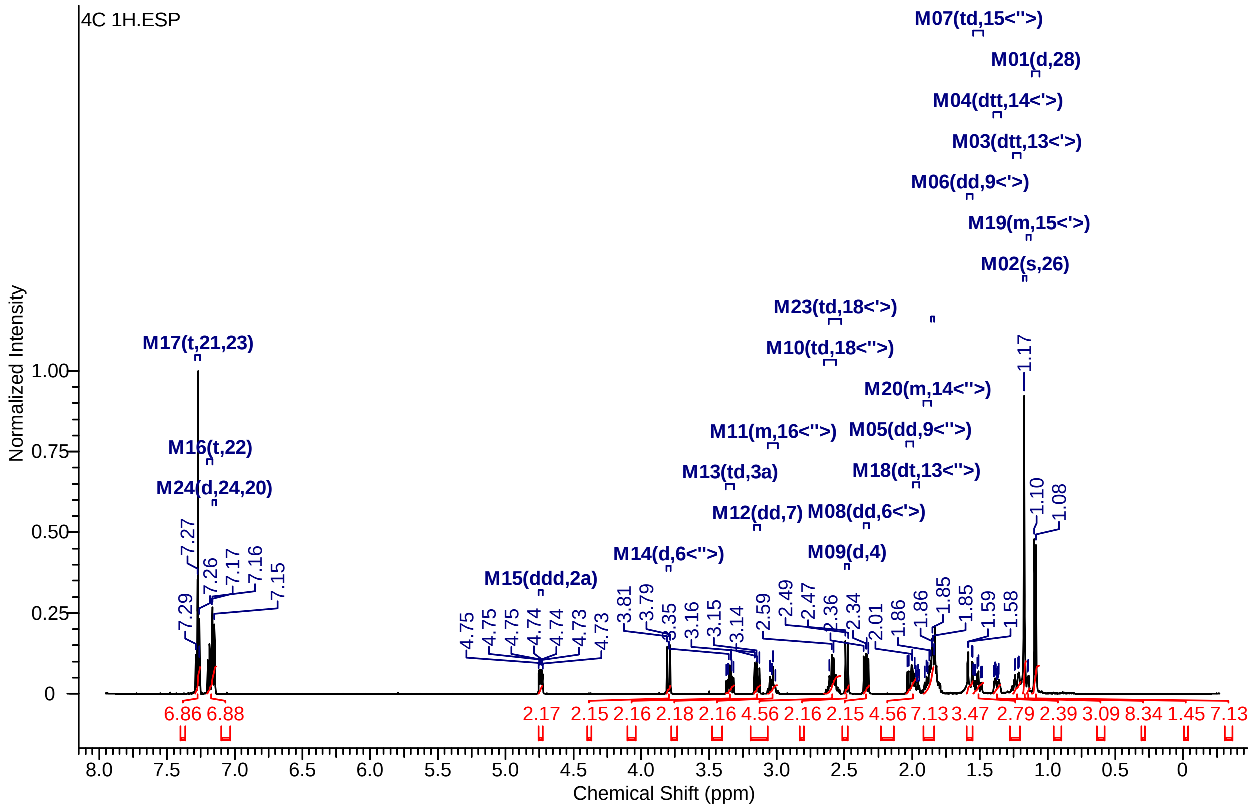
4C 1H.ESP
Normalized Intensity
Chemical Shift (ppm)
M17(t,21,23)
M16(t,22)
M24(d,24,20)
M15(ddd,2a)
M14(d,6<">)
M13(td,3a)
M12(dd,7)
M11(m,16<">)
M10(td,18<">)
M23(td,18<'>)
M09(d,4)
M08(dd,6<'>)
M18(dt,13<">)
M05(dd,9<">)
M20(m,14<">)
M02(s,26)
M19(m,15<'>)
M06(dd,9<'>)
M03(dtt,13<'>)
M04(dtt,14<'>)
M01(d,28)
M07(td,15<">)
6.86 6.88 2.17 2.15 2.16 2.18 2.16 4.56 2.16 2.15 4.56 7.13 3.47 2.79 2.39 3.09 8.34 1.45 7.13

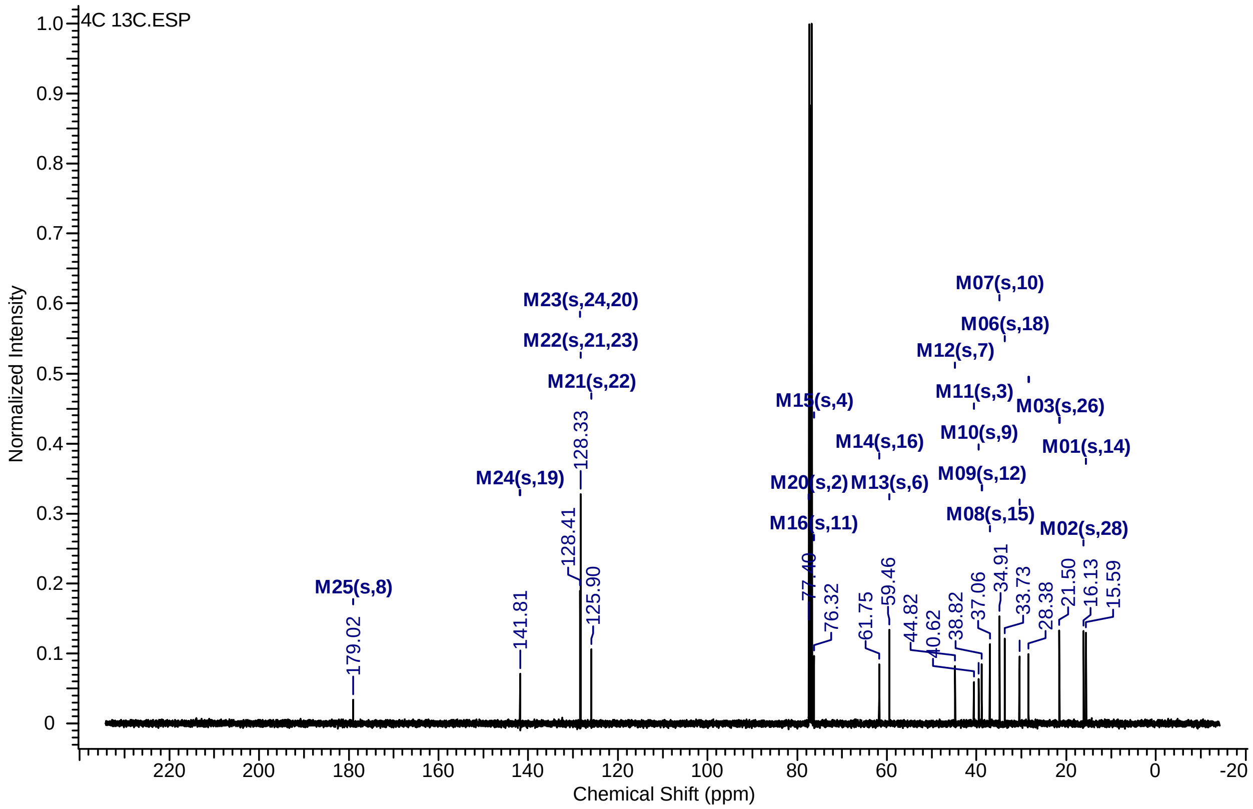
4C 13C.ESP
Normalized Intensity
Chemical Shift (ppm)
M25(s,8)
M24(s,19)
M23(s,24,20)
M22(s,21,23)
M21(s,22)
M15(s,4)
M20(s,2)
M16(s,11)
M14(s,16)
M13(s,6)
M12(s,7)
M11(s,3)
M10(s,9)
M09(s,12)
M08(s,15)
M07(s,10)
M06(s,18)
M03(s,26)
M01(s,14)
M02(s,28)
179.02
141.81
128.41
128.33
125.90
77.40
76.32
61.75
59.46
44.82
40.62
38.82
37.06
34.91
33.73
28.38
21.50
16.13
15.59

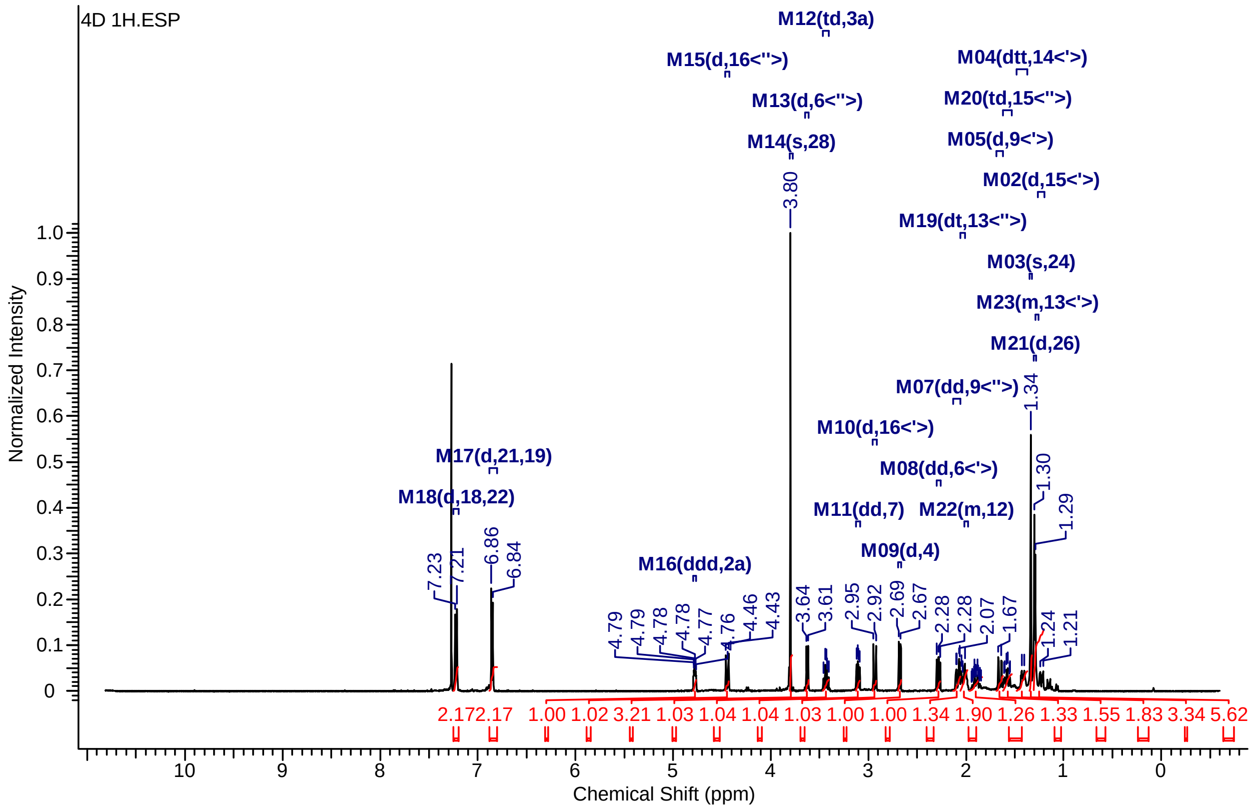

4D 1H.ESP
M12(td,3a)
M15(d,16<''>)
M04(dtt,14<'>)
M13(d,6<''>)
M20(td,15<''>)
M14(s,28)
M05(d,9<'>)
M02(d,15<'>)
M19(dt,13<''>)
M03(s,24)
M23(m,13<'>)
M21(d,26)
M07(dd,9<''>)
M10(d,16<'>)
M17(d,21,19)
M08(dd,6<'>)
M18(d,18,22)
M11(dd,7)
M22(m,12)
M09(d,4)
M16(ddd,2a)
7.23 7.21 6.86 6.84 4.79 4.79 4.78 4.78 4.77 4.76 4.46 4.43 3.80 3.64 3.61 2.95 2.92 2.69 2.67 2.28 2.28 2.07 1.67 1.34 1.30 1.29 1.24 1.21
2.17 2.17 1.00 1.02 3.21 1.03 1.04 1.04 1.03 1.00 1.00 1.34 1.90 1.26 1.33 1.55 1.83 3.34 5.62
Normalized Intensity
Chemical Shift (ppm)

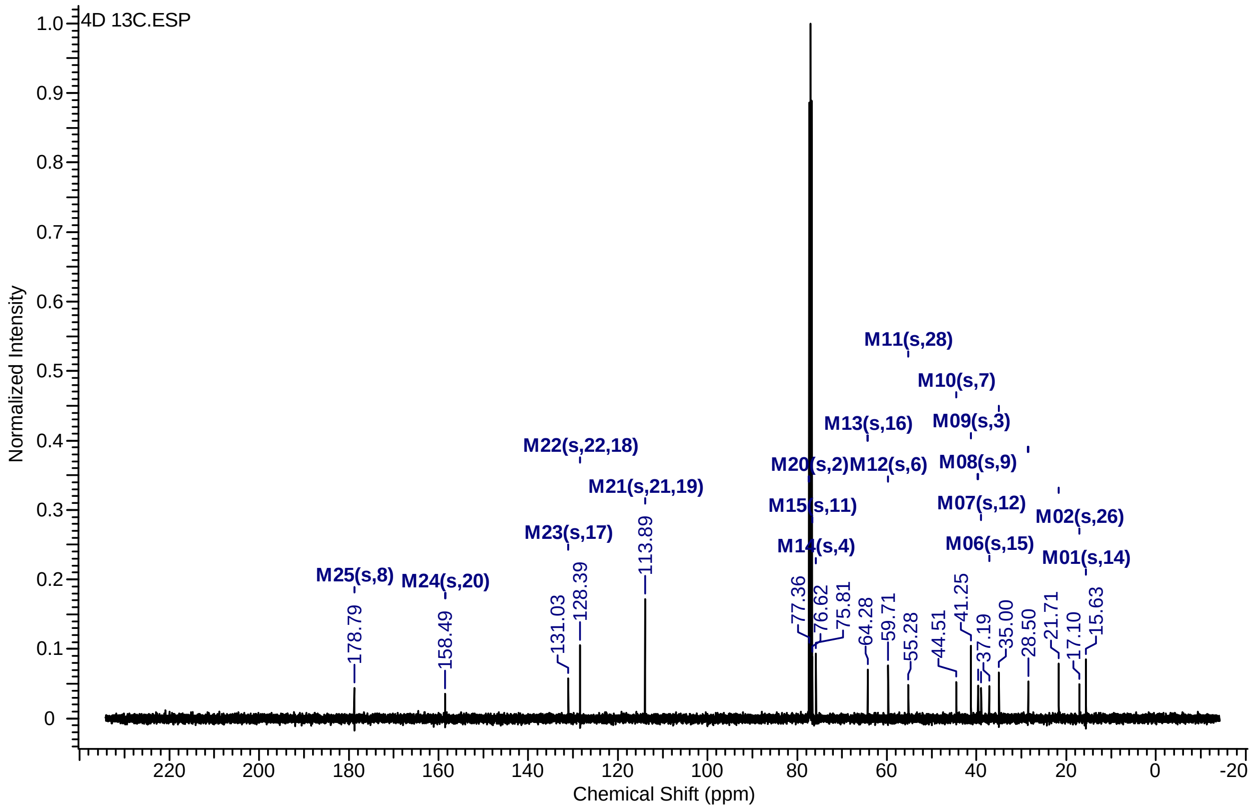

4D 13C.ESP
Normalized Intensity
Chemical Shift (ppm)
M25(s,8)
M24(s,20)
M23(s,17)
M22(s,22,18)
M21(s,21,19)
M20(s,2)
M15(s,11)
M14(s,4)
M13(s,16)
M12(s,6)
M11(s,28)
M10(s,7)
M09(s,3)
M08(s,9)
M07(s,12)
M06(s,15)
M02(s,26)
M01(s,14)
178.79
158.49
131.03
128.39
113.89
77.36
76.62
75.81
64.28
59.71
55.28
44.51
41.25
37.19
35.00
28.50
21.71
17.10
15.63

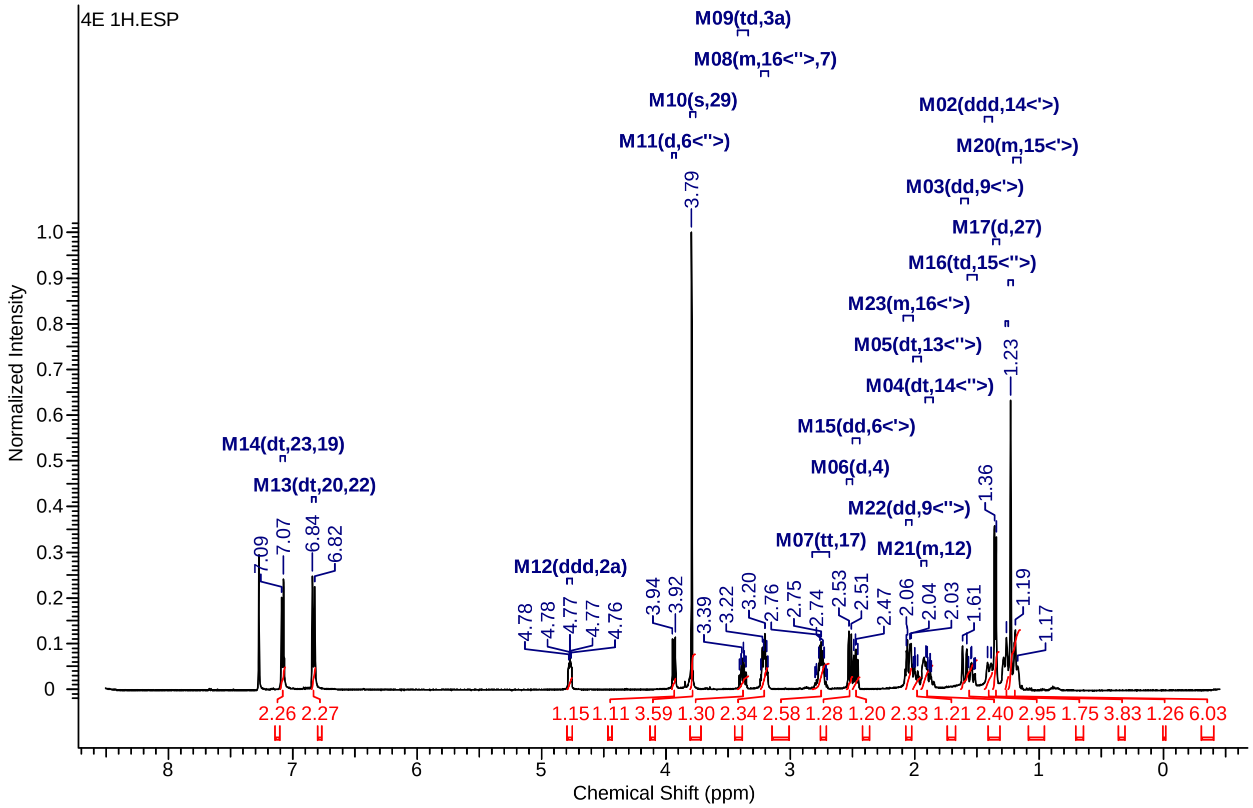
4E 1H.ESP
Normalized Intensity
Chemical Shift (ppm)
M14(dt,23,19)
M13(dt,20,22)
M12(ddd,2a)
M11(d,6<">)
M10(s,29)
M09(td,3a)
M08(m,16<">,7)
M07(tt,17)
M06(d,4)
M15(dd,6<'>)
M22(dd,9<">)
M21(m,12)
M04(dt,14<">)
M05(dt,13<">)
M23(m,16<'>)
M16(td,15<">)
M17(d,27)
M03(dd,9<'>)
M20(m,15<'>)
M02(ddd,14<'>)
2.26 2.27 1.15 1.11 3.59 1.30 2.34 2.58 1.28 1.20 2.33 1.21 2.40 2.95 1.75 3.83 1.26 6.03

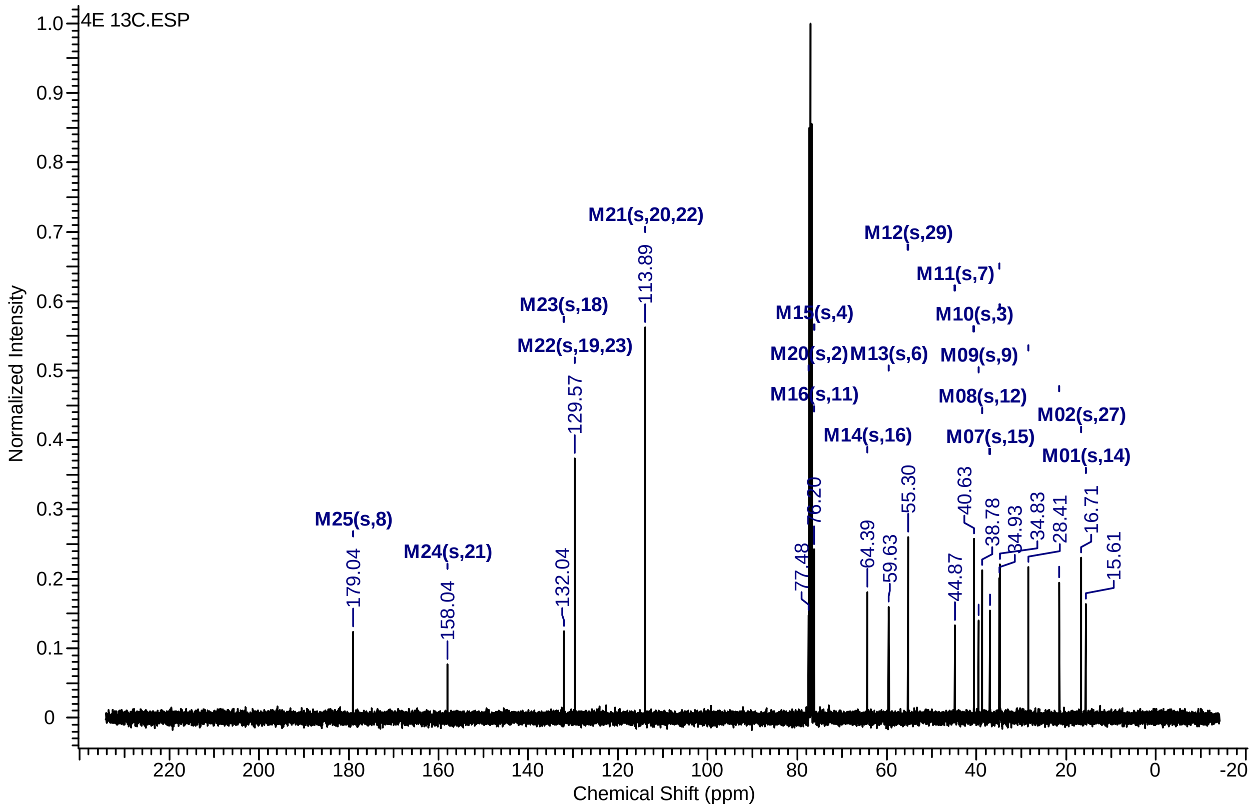

4E 13C.ESP
Normalized Intensity
Chemical Shift (ppm)
M25(s,8)
179.04
M24(s,21)
158.04
M23(s,18)
132.04
M22(s,19,23)
129.57
M21(s,20,22)
113.89
M15(s,4)
M20(s,2)
M16(s,11)
77.48
76.20
M14(s,16)
64.39
M13(s,6)
59.63
M12(s,29)
55.30
M11(s,7)
44.87
M10(s,3)
40.63
M09(s,9)
38.78
M08(s,12)
M07(s,15)
34.93
34.83
28.41
M02(s,27)
16.71
M01(s,14)
15.61

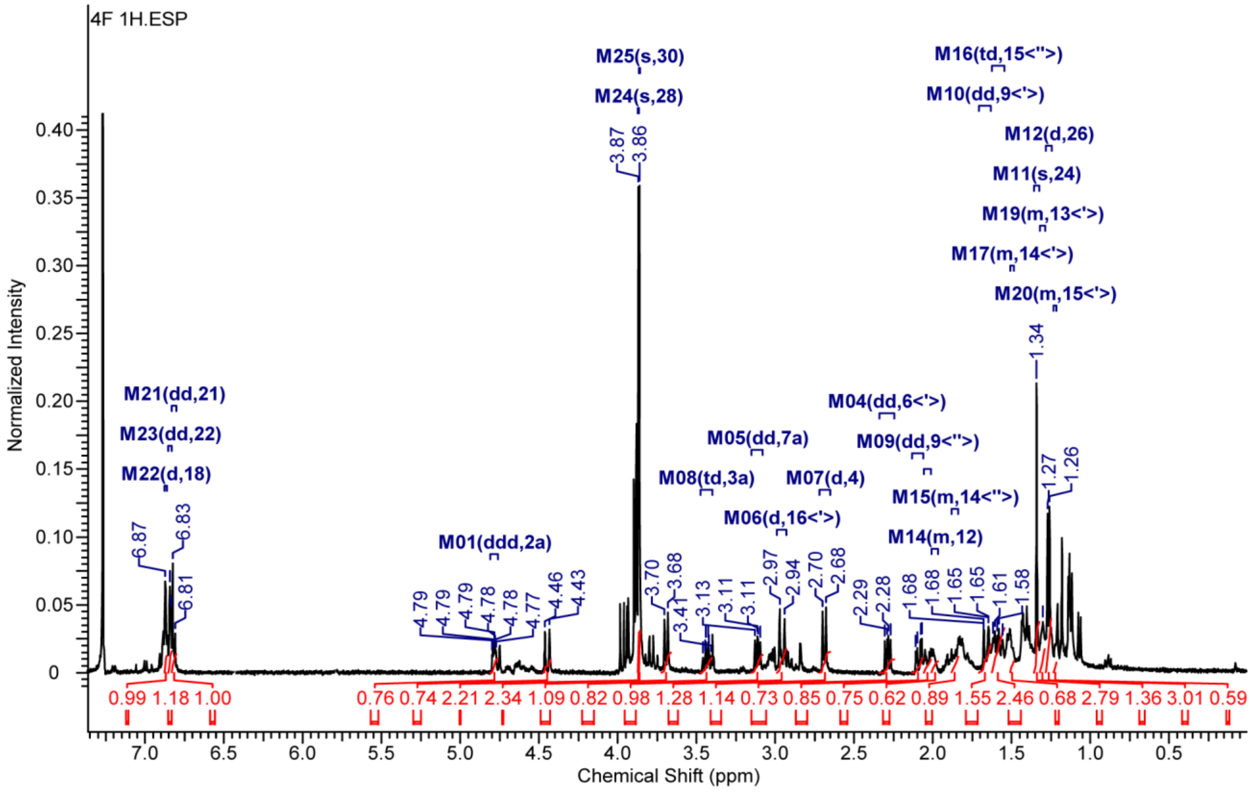
4F 1H.ESP
Normalized Intensity
Chemical Shift (ppm)
M25(s,30)
M24(s,28)
M16(td,15<''>)
M10(dd,9<'>)
M12(d,26)
M11(s,24)
M19(m,13<'>)
M17(m,14<'>)
M20(m,15<'>)
M21(dd,21)
M23(dd,22)
M22(d,18)
M04(dd,6<'>)
M05(dd,7a)
M09(dd,9<''>)
M08(td,3a)
M07(d,4)
M15(m,14<''>)
M06(d,16<'>)
M01(ddd,2a)
M14(m,12)

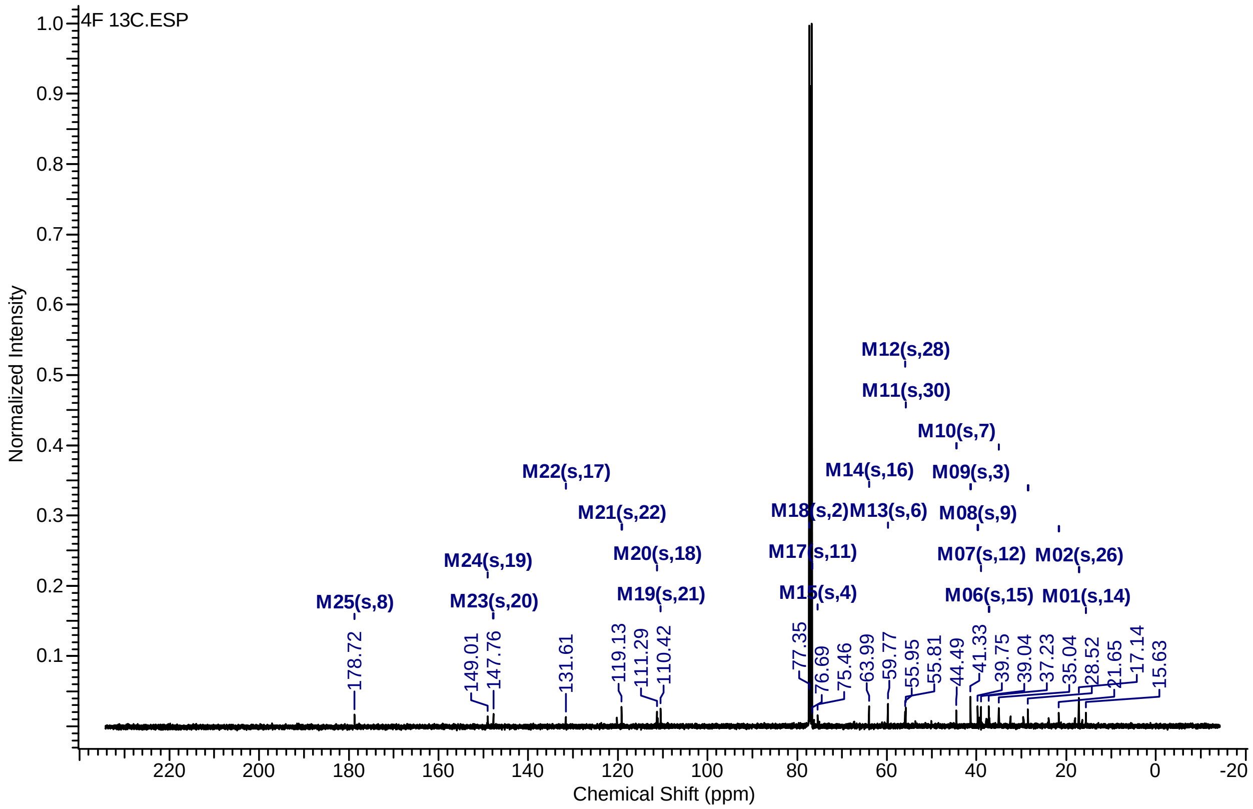
4F 13C.ESP
Normalized Intensity
Chemical Shift (ppm)
M25(s,8)
M24(s,19)
M23(s,20)
M22(s,17)
M21(s,22)
M20(s,18)
M19(s,21)
M18(s,2)
M17(s,11)
M16(s,4)
M14(s,16)
M13(s,6)
M12(s,28)
M11(s,30)
M10(s,7)
M09(s,3)
M08(s,9)
M07(s,12)
M06(s,15)
M02(s,26)
M01(s,14)
178.72
149.01
147.76
131.61
119.13
111.29
110.42
77.35
76.69
75.46
63.99
59.77
55.95
55.81
44.49
41.33
39.75
39.04
37.23
35.04
28.52
21.65
17.14
15.63

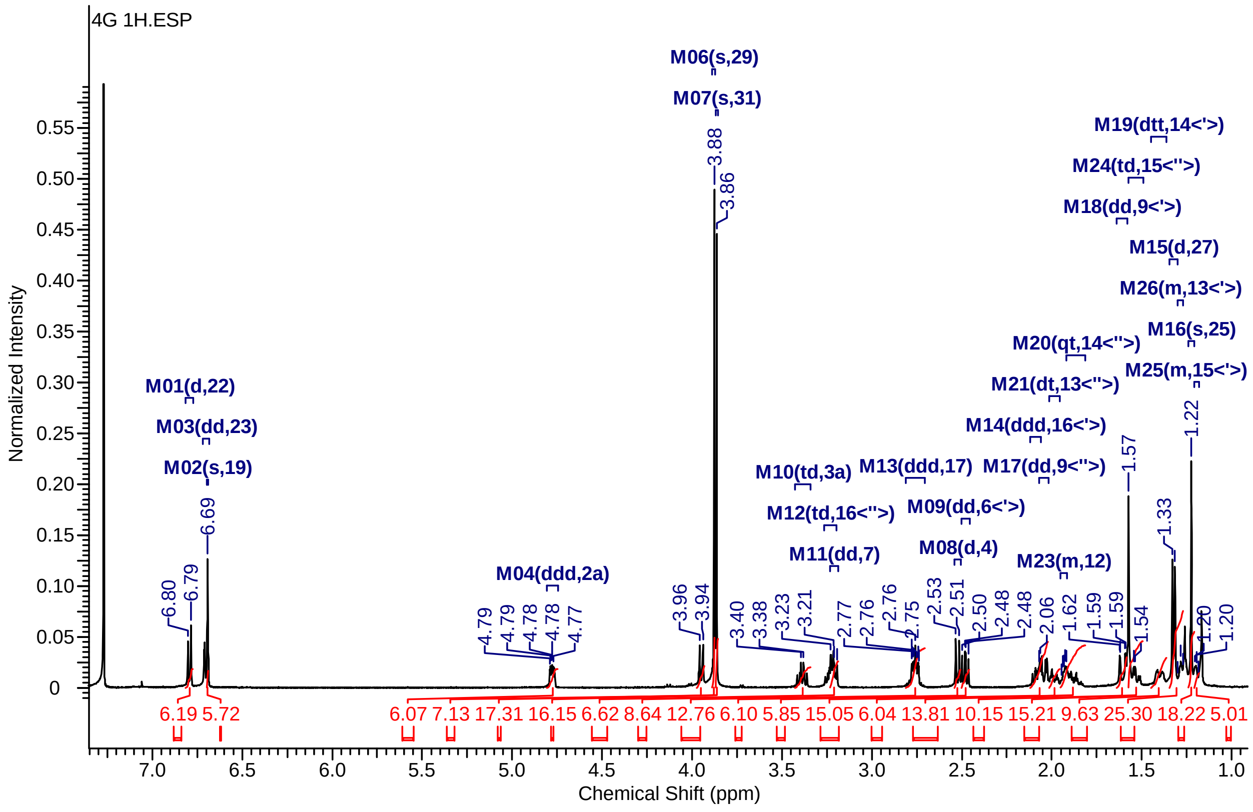

4G 1H.ESP
M06(s,29)
M07(s,31)
M19(dtt,14<'>)
M24(td,15<''>)
M18(dd,9<'>)
M15(d,27)
M26(m,13<'>)
M16(s,25)
M20(qt,14<''>)
M25(m,15<'>)
M21(dt,13<''>)
M01(d,22)
M03(dd,23)
M02(s,19)
M14(ddd,16<'>)
M10(td,3a)
M13(ddd,17)
M17(dd,9<''>)
M12(td,16<''>)
M09(dd,6<'>)
M11(dd,7)
M08(d,4)
M23(m,12)
M04(ddd,2a)
Normalized Intensity
Chemical Shift (ppm)
6.19 5.72 6.07 7.13 17.31 16.15 6.62 8.64 12.76 6.10 5.85 15.05 6.04 13.81 10.15 15.21 9.63 25.30 18.22 5.01

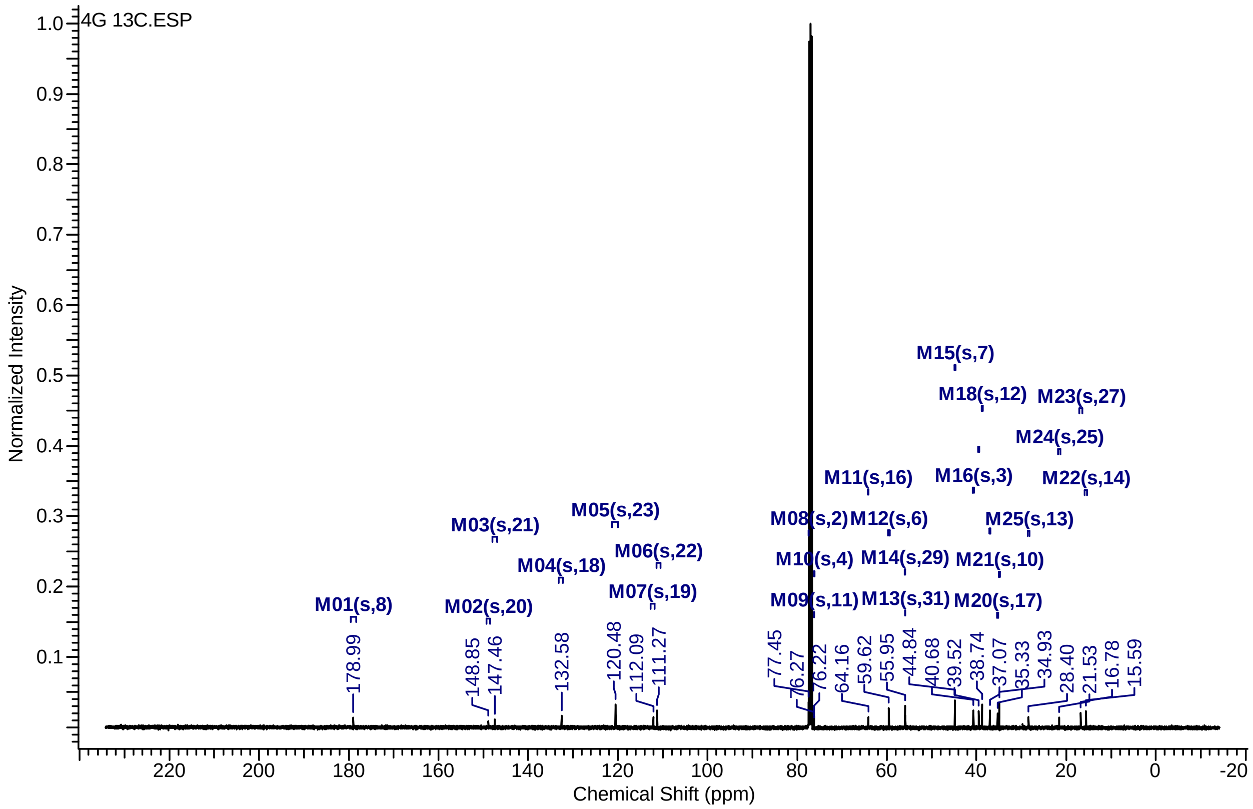
4G 13C.ESP
Normalized Intensity
Chemical Shift (ppm)
M01(s,8)
M02(s,20)
M03(s,21)
M04(s,18)
M05(s,23)
M06(s,22)
M07(s,19)
M08(s,2)
M09(s,11)
M10(s,4)
M11(s,16)
M12(s,6)
M13(s,31)
M14(s,29)
M15(s,7)
M16(s,3)
M18(s,12)
M20(s,17)
M21(s,10)
M22(s,14)
M23(s,27)
M24(s,25)
M25(s,13)
178.99
148.85
147.46
132.58
120.48
112.09
111.27
77.45
76.27
76.22
64.16
59.62
55.95
44.84
40.68
39.52
38.74
37.07
35.33
34.93
28.40
21.53
16.78
15.59

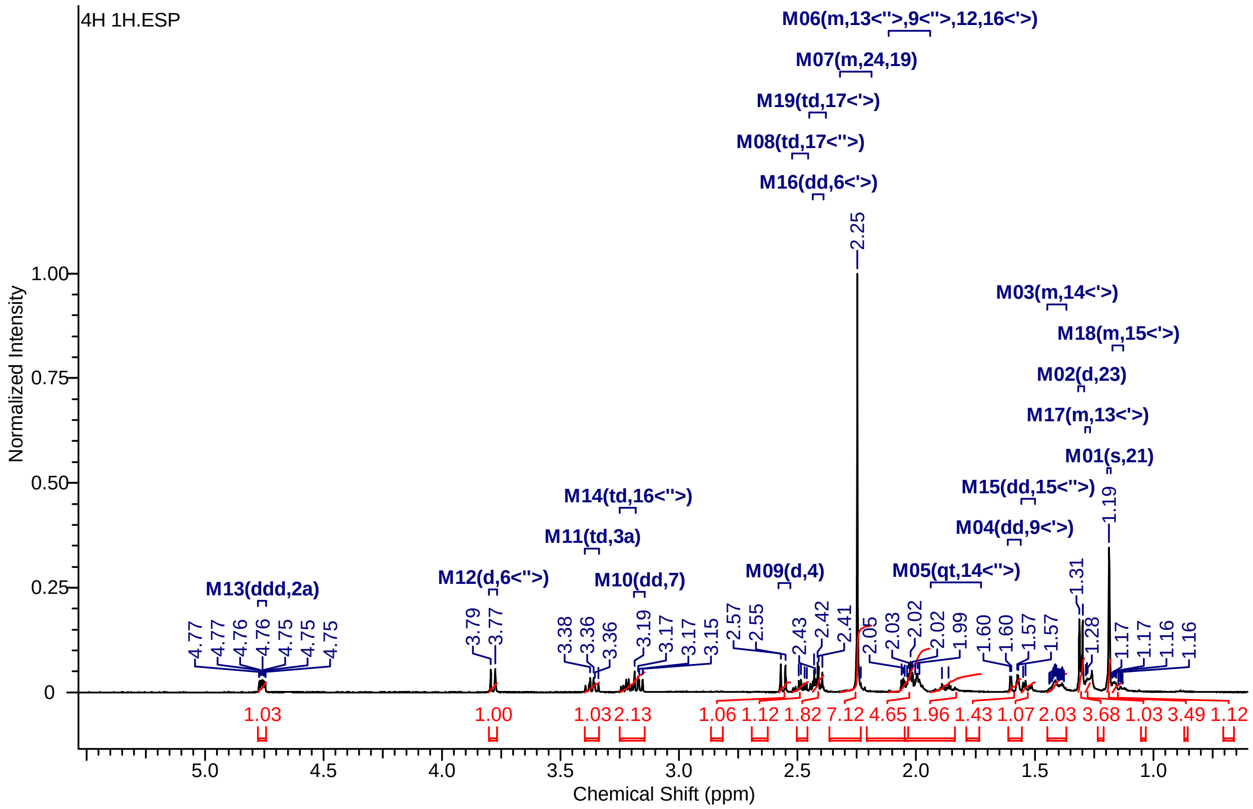

4H 1H.ESP
M06(m,13<">,9<">,12,16<'>)
M07(m,24,19)
M19(td,17<'>)
M08(td,17<">)
M16(dd,6<'>)
M03(m,14<'>)
M18(m,15<'>)
M02(d,23)
M17(m,13<'>)
M01(s,21)
M15(dd,15<">)
M04(dd,9<'>)
M14(td,16<">)
M11(td,3a)
M12(d,6<">)
M10(dd,7)
M09(d,4)
M05(qt,14<">)
M13(ddd,2a)
Normalized Intensity
Chemical Shift (ppm)

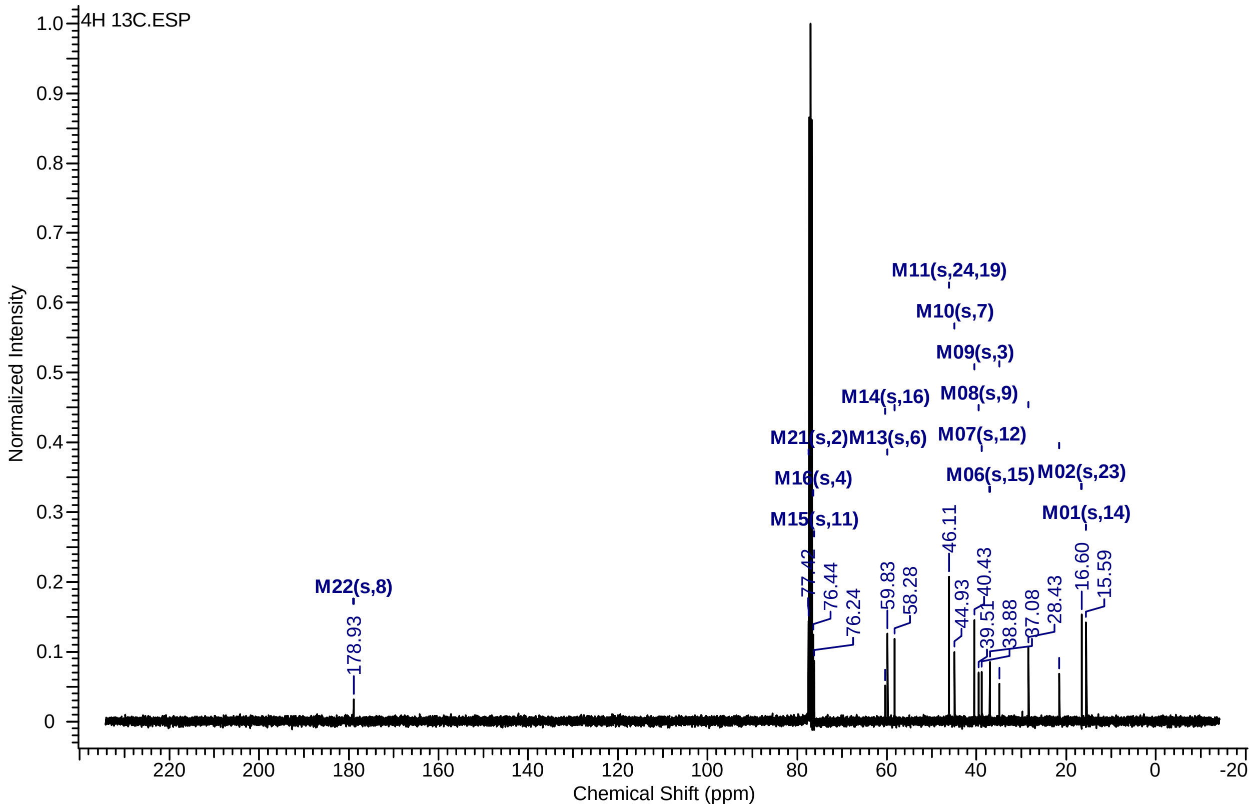

4H 13C.ESP
Normalized Intensity
Chemical Shift (ppm)
M22(s,8)
178.93
M21(s,2)
M16(s,4)
M15(s,11)
77.42
76.44
76.24
M14(s,16)
M13(s,6)
59.83
58.28
M11(s,24,19)
M10(s,7)
M09(s,3)
M08(s,9)
M07(s,12)
M06(s,15)
46.11
44.93
40.43
39.51
38.88
37.08
28.43
M02(s,23)
M01(s,14)
16.60
15.59

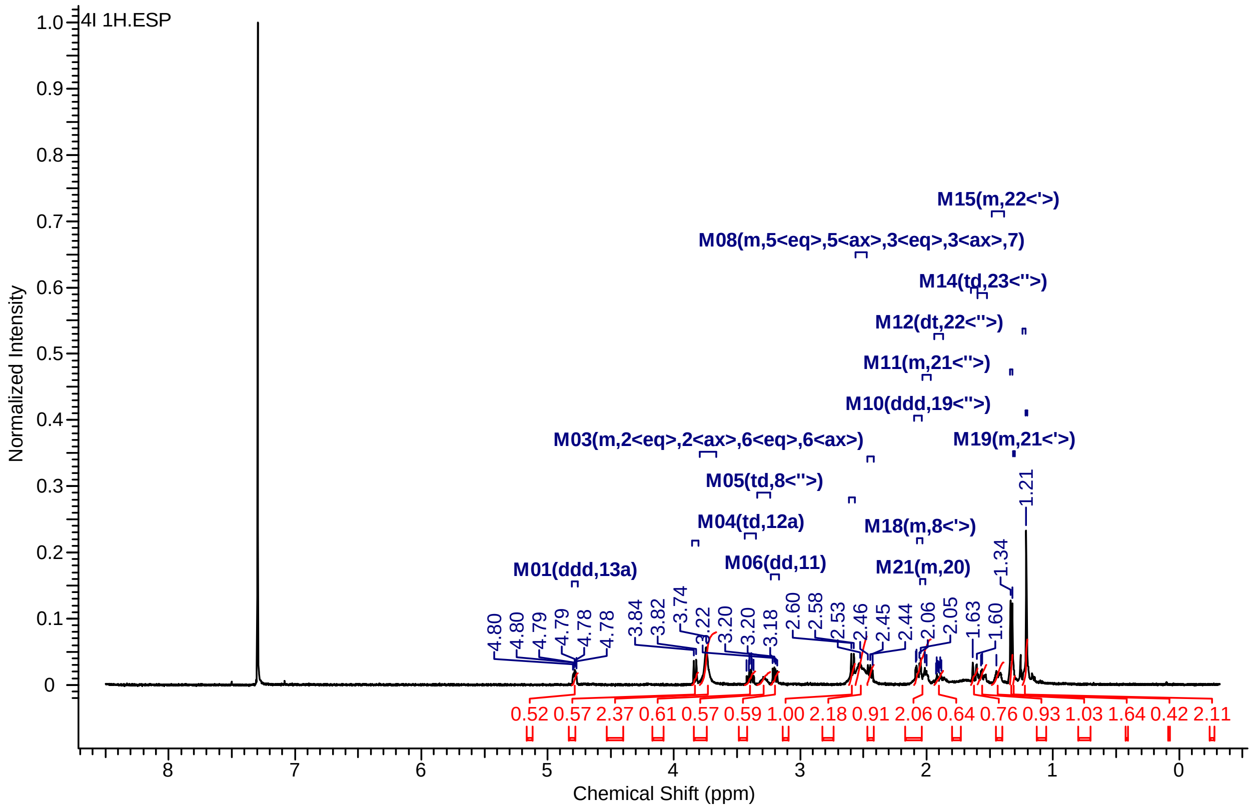
4I 1H.ESP
Normalized Intensity
Chemical Shift (ppm)
M15(m,22<'>)
M08(m,5<eq>,5<ax>,3<eq>,3<ax>,7)
M14(td,23<''>)
M12(dt,22<''>)
M11(m,21<''>)
M10(ddd,19<''>)
M03(m,2<eq>,2<ax>,6<eq>,6<ax>)
M19(m,21<'>)
M05(td,8<''>)
M04(td,12a)
M18(m,8<'>)
M01(ddd,13a)
M06(dd,11)
M21(m,20)
4.80 4.80 4.79 4.79 4.78 4.78 3.84 3.82 3.74 3.22 3.20 3.20 3.18 2.60 2.58 2.53 2.46 2.45 2.44 2.06 2.05 1.63 1.60 1.34 1.21
0.52 0.57 2.37 0.61 0.57 0.59 1.00 2.18 0.91 2.06 0.64 0.76 0.93 1.03 1.64 0.42 2.11

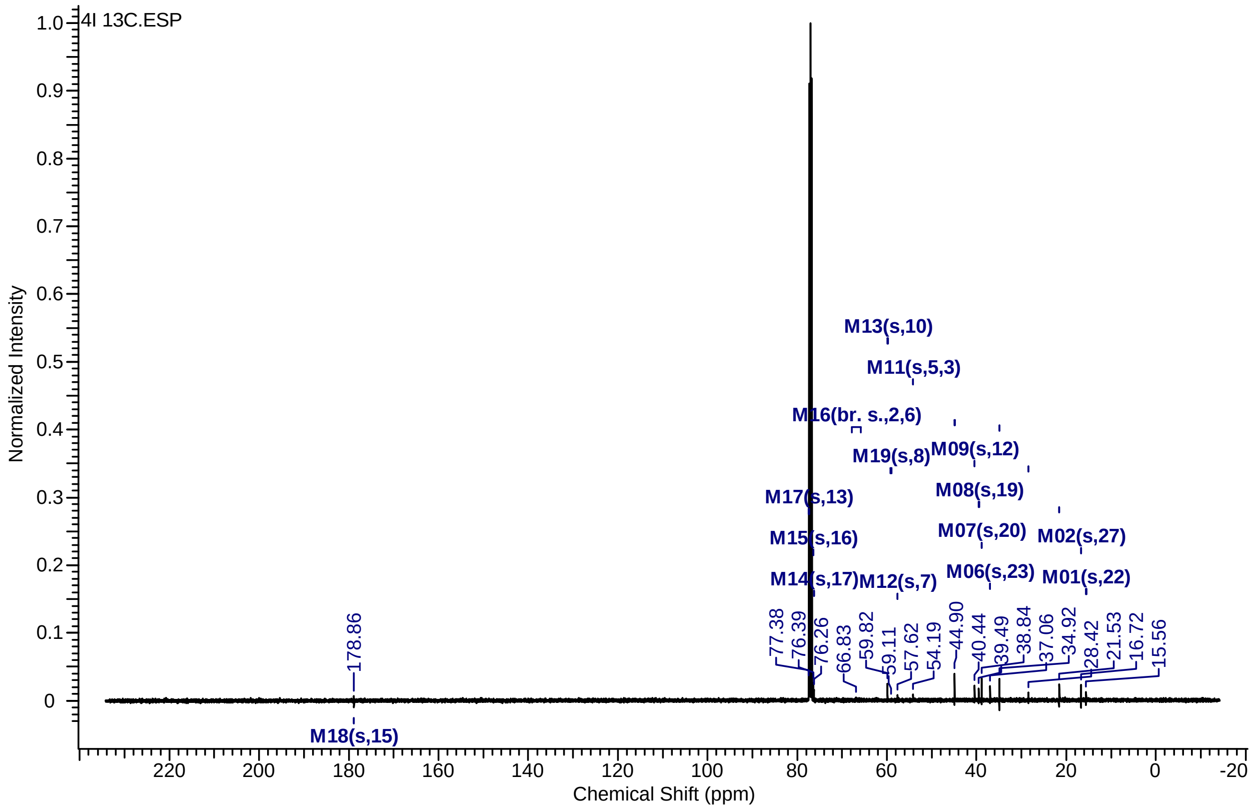

4I 13C.ESP
Normalized Intensity
Chemical Shift (ppm)
178.86
M18(s,15)
77.38
76.39
76.26
66.83
59.82
59.11
57.62
54.19
44.90
40.44
39.49
38.84
37.06
34.92
28.42
21.53
16.72
15.56
M13(s,10)
M11(s,5,3)
M16(br. s.,2,6)
M19(s,8)
M09(s,12)
M17(s,13)
M08(s,19)
M15(s,16)
M07(s,20)
M02(s,27)
M14(s,17)
M12(s,7)
M06(s,23)
M01(s,22)

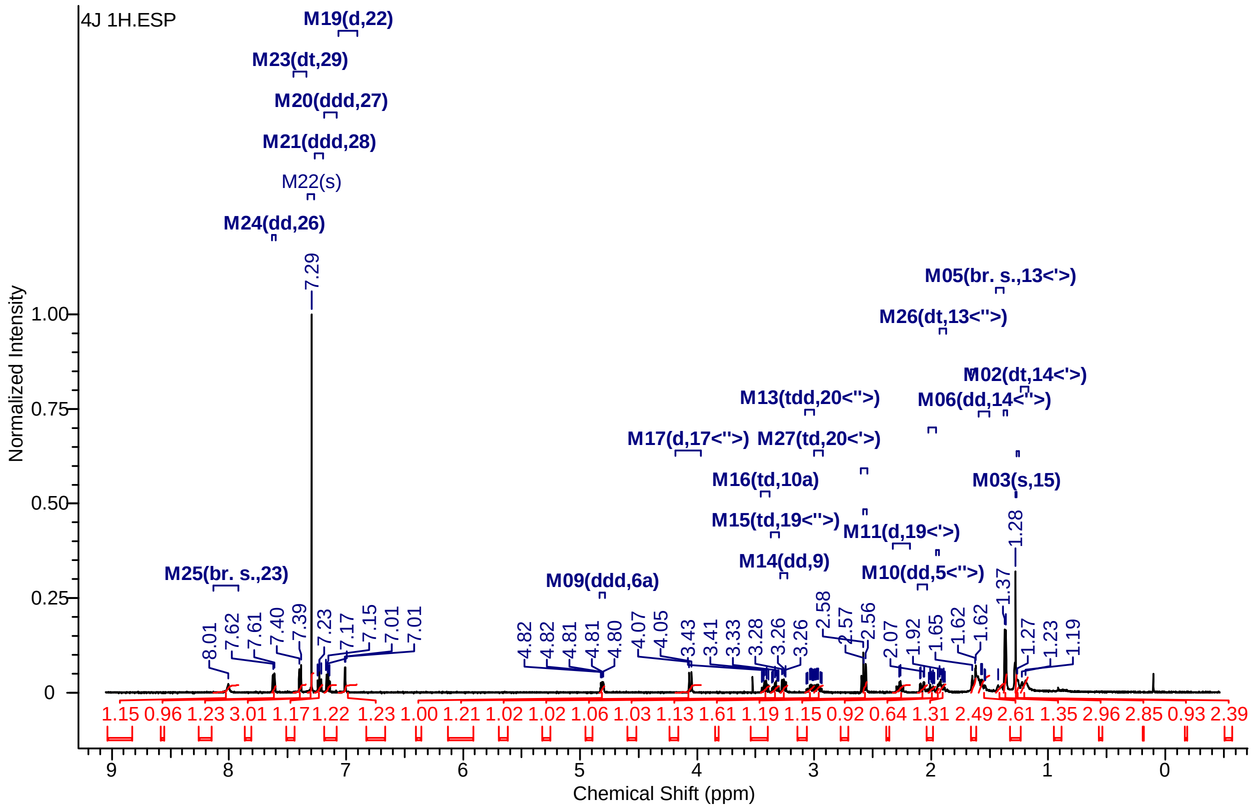
4J 1H.ESP
Normalized Intensity
Chemical Shift (ppm)
M19(d,22)
M23(dt,29)
M20(ddd,27)
M21(ddd,28)
M22(s)
M24(dd,26)
M25(br. s.,23)
M09(ddd,6a)
M17(d,17<''>)
M13(tdd,20<''>)
M27(td,20<'>)
M16(td,10a)
M15(td,19<''>)
M14(dd,9)
M11(d,19<'>)
M10(dd,5<''>)
M05(br. s.,13<'>)
M26(dt,13<''>)
M02(dt,14<'>)
M06(dd,14<''>)
M03(s,15)

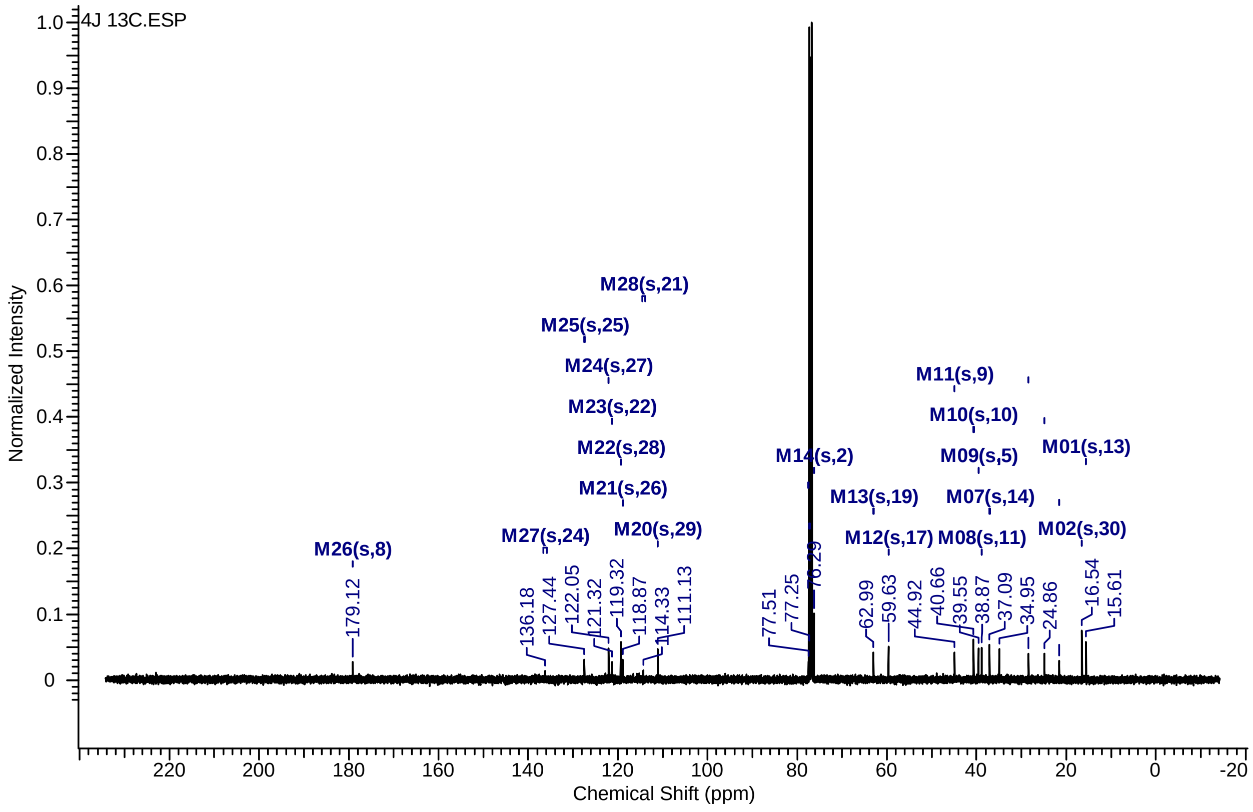

4J 13C.ESP
Normalized Intensity
Chemical Shift (ppm)
M26(s,8)
M27(s,24)
M28(s,21)
M25(s,25)
M24(s,27)
M23(s,22)
M22(s,28)
M21(s,26)
M20(s,29)
M14(s,2)
M13(s,19)
M12(s,17)
M11(s,9)
M10(s,10)
M09(s,5)
M07(s,14)
M08(s,11)
M01(s,13)
M02(s,30)
179.12
136.18
127.44
122.05
121.32
119.32
118.87
114.33
111.13
77.51
77.25
76.99
62.99
59.63
44.92
40.66
39.55
38.87
37.09
34.95
24.86
16.54
15.61

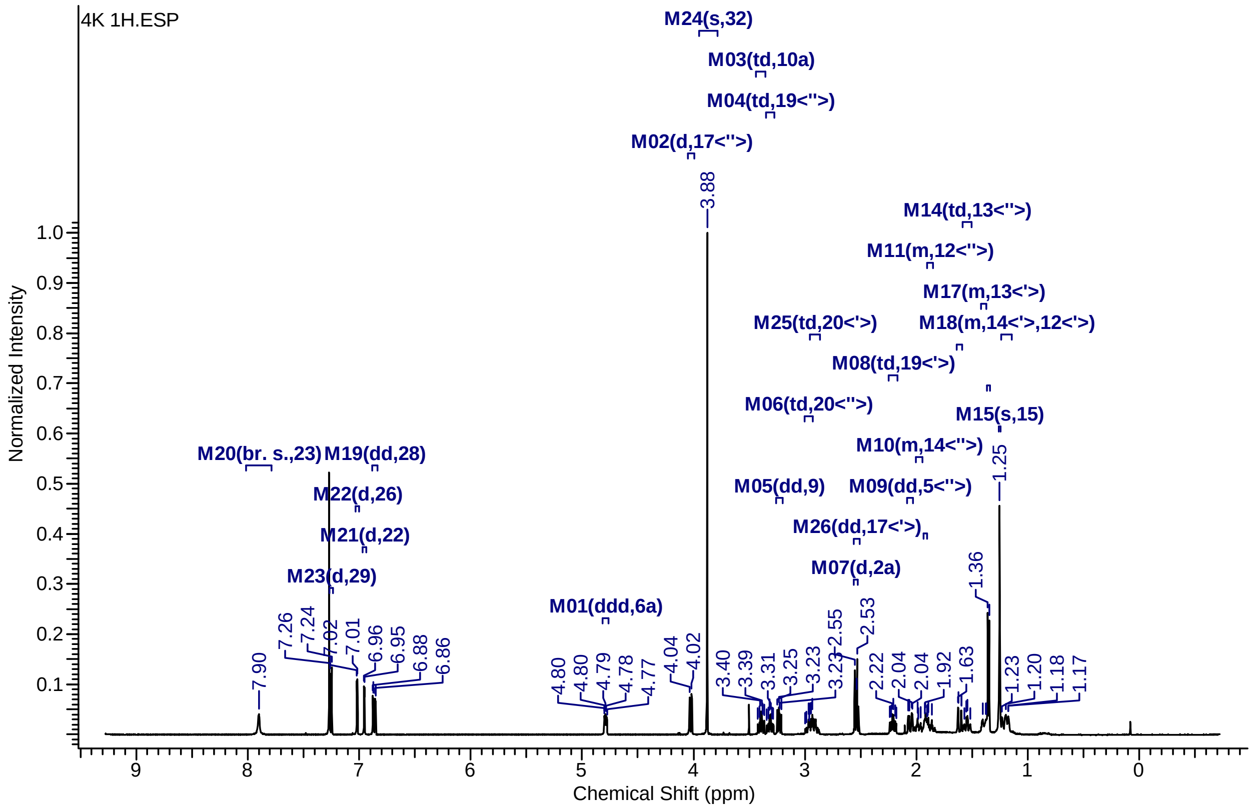
4K 1H.ESP
M24(s,32)
M03(td,10a)
M04(td,19<">)
M02(d,17<">)
M14(td,13<">)
M11(m,12<">)
M17(m,13<'>)
M25(td,20<'>)
M18(m,14<'>,12<'>)
M08(td,19<'>)
M06(td,20<">)
M15(s,15)
M10(m,14<">)
M20(br. s.,23)
M19(dd,28)
M22(d,26)
M21(d,22)
M23(d,29)
M05(dd,9)
M09(dd,5<">)
M26(dd,17<'>)
M07(d,2a)
M01(ddd,6a)
3.88
1.25
1.36
7.90
7.26
7.24
7.02
7.01
6.96
6.95
6.88
6.86
4.80
4.80
4.79
4.78
4.77
4.04
4.02
3.40
3.39
3.31
3.25
3.23
3.23
2.55
2.53
2.22
2.04
2.04
1.92
1.63
1.23
1.20
1.18
1.17
Normalized Intensity
Chemical Shift (ppm)

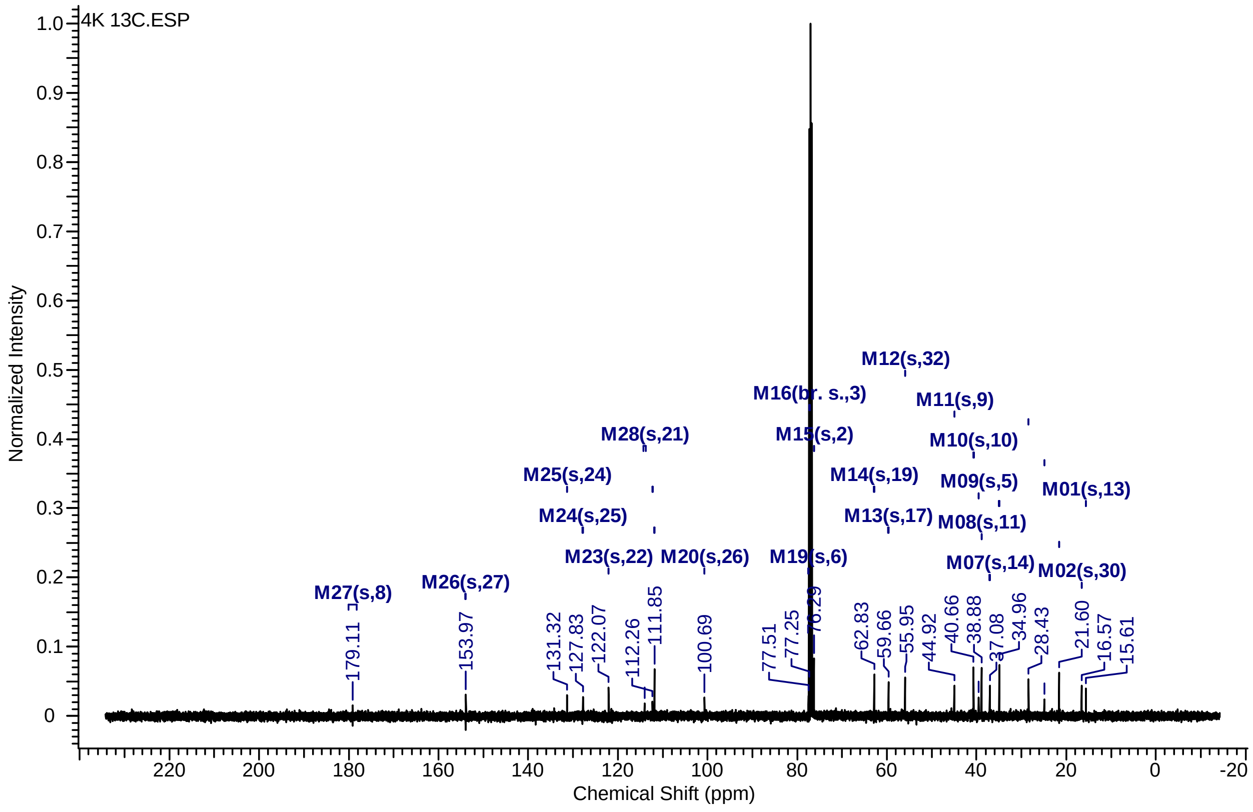
4K 13C.ESP
Normalized Intensity
Chemical Shift (ppm)
M27(s,8)
M26(s,27)
M25(s,24)
M24(s,25)
M23(s,22)
M28(s,21)
M20(s,26)
M16(br. s.,3)
M15(s,2)
M19(s,6)
M12(s,32)
M11(s,9)
M10(s,10)
M14(s,19)
M13(s,17)
M09(s,5)
M08(s,11)
M07(s,14)
M01(s,13)
M02(s,30)
179.11
153.97
131.32
127.83
122.07
112.26
111.85
100.69
77.51
77.25
76.99
62.83
59.66
55.95
44.92
40.66
38.88
37.08
34.96
28.43
21.60
16.57
15.61

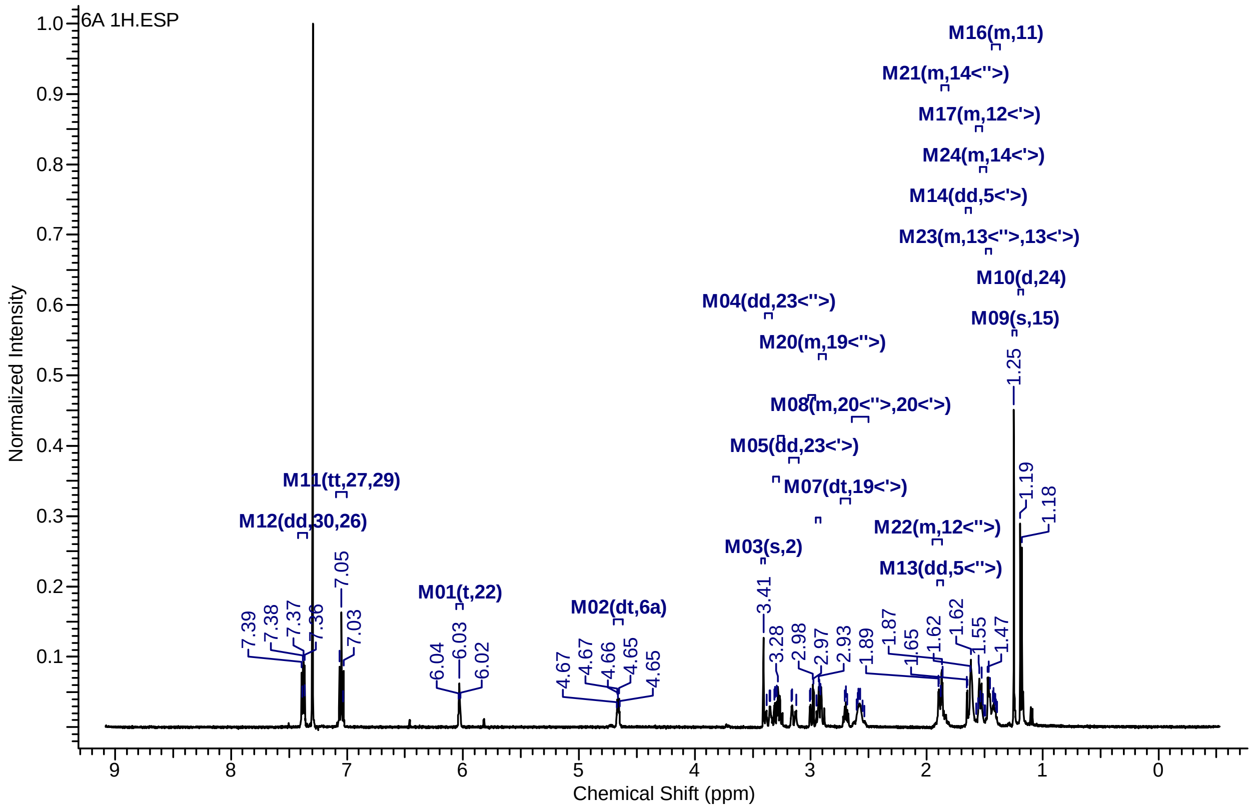
6A 1H.ESP
Normalized Intensity
Chemical Shift (ppm)

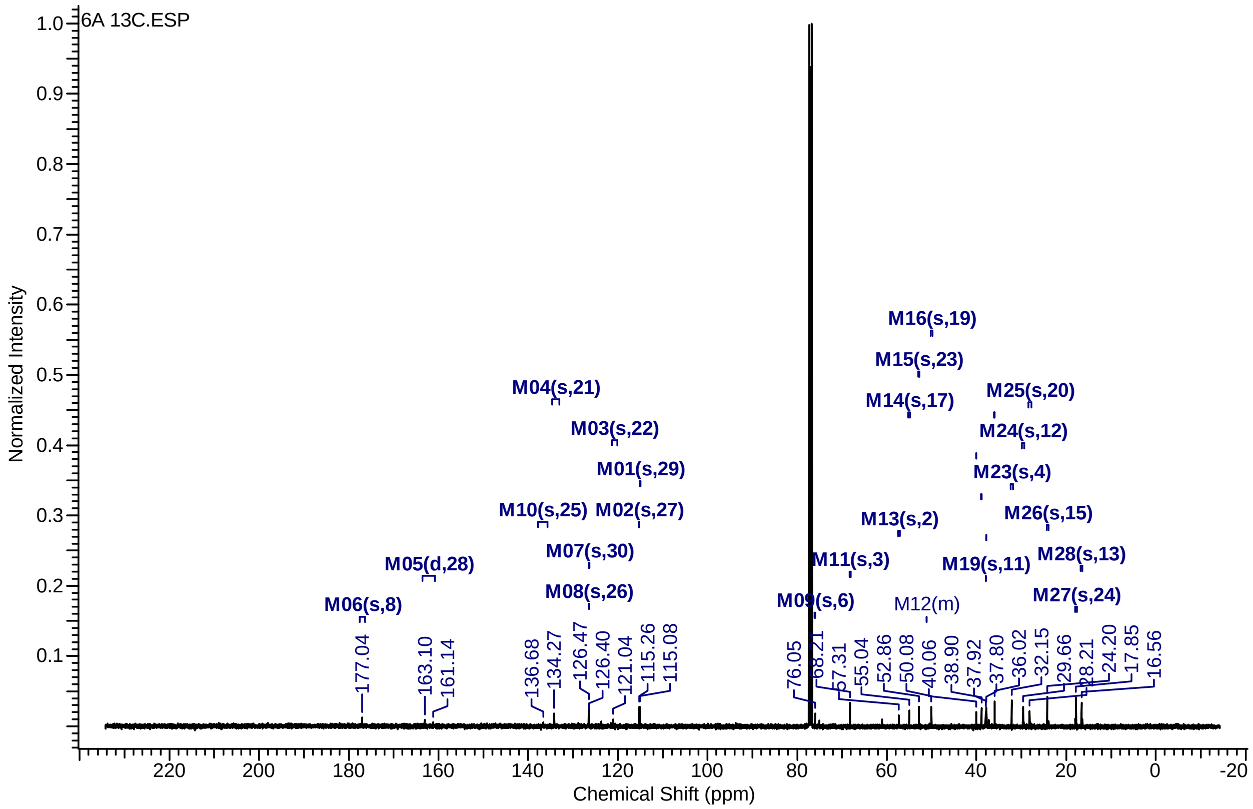
6A 13C.ESP
Normalized Intensity
Chemical Shift (ppm)
M01(s,29)
M02(s,27)
M03(s,22)
M04(s,21)
M05(d,28)
M06(s,8)
M07(s,30)
M08(s,26)
M09(s,6)
M10(s,25)
M11(s,3)
M12(m)
M13(s,2)
M14(s,17)
M15(s,23)
M16(s,19)
M19(s,11)
M23(s,4)
M24(s,12)
M25(s,20)
M26(s,15)
M27(s,24)
M28(s,13)
177.04
163.10
161.14
136.68
134.27
126.47
126.40
121.04
115.26
115.08
76.05
68.21
57.31
55.04
52.86
50.08
40.06
38.90
37.92
37.80
36.02
32.15
29.66
28.21
24.20
17.85
16.56

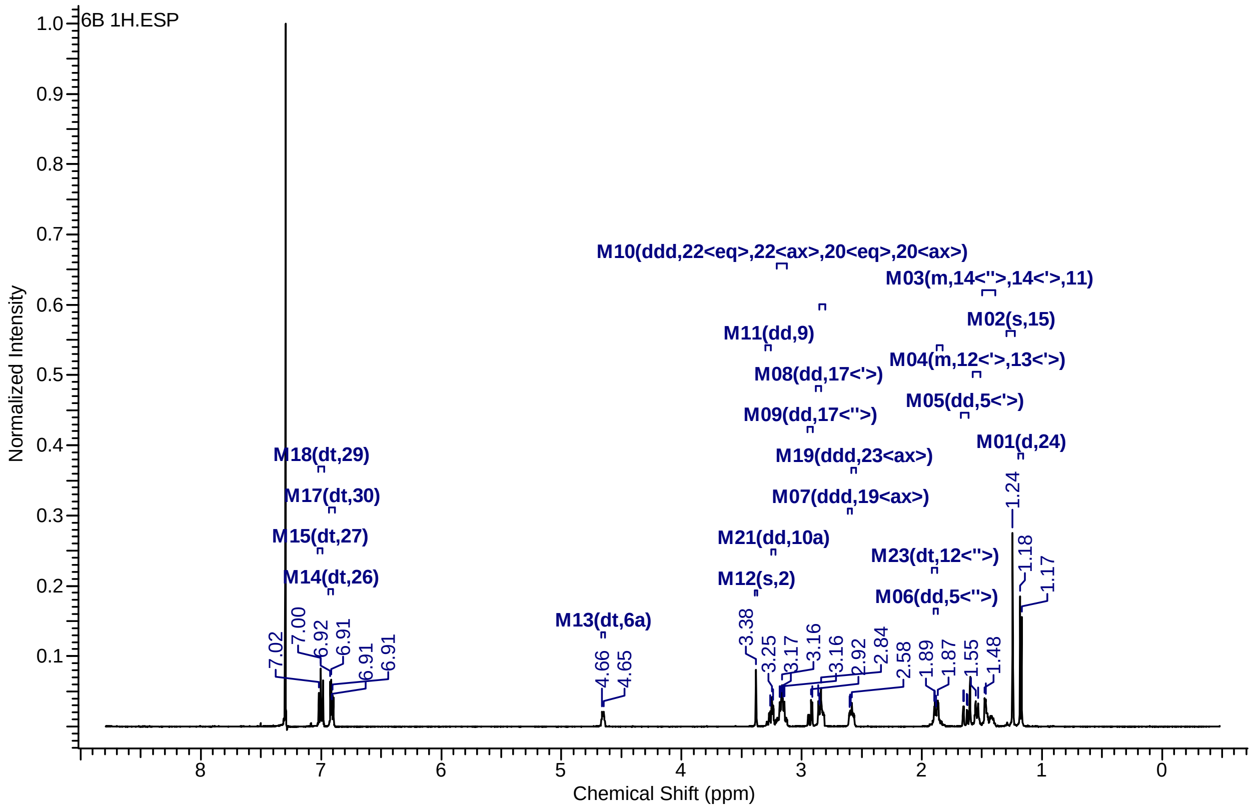

6B 1H.ESP
Normalized Intensity
Chemical Shift (ppm)
M10(ddd,22<eq>,22<ax>,20<eq>,20<ax>)
M03(m,14<''>,14<'>,11)
M02(s,15)
M11(dd,9)
M04(m,12<'>,13<'>)
M08(dd,17<'>)
M05(dd,5<'>)
M09(dd,17<''>)
M01(d,24)
M18(dt,29)
M19(ddd,23<ax>)
M17(dt,30)
M07(ddd,19<ax>)
M15(dt,27)
M21(dd,10a)
M23(dt,12<''>)
M14(dt,26)
M12(s,2)
M06(dd,5<''>)
M13(dt,6a)
7.02 7.00 6.92 6.91 6.91 6.91
4.66 4.65
3.38 3.25 3.17 3.16 3.16 2.92 2.84 2.58
1.89 1.87 1.55 1.48 1.24 1.18 1.17

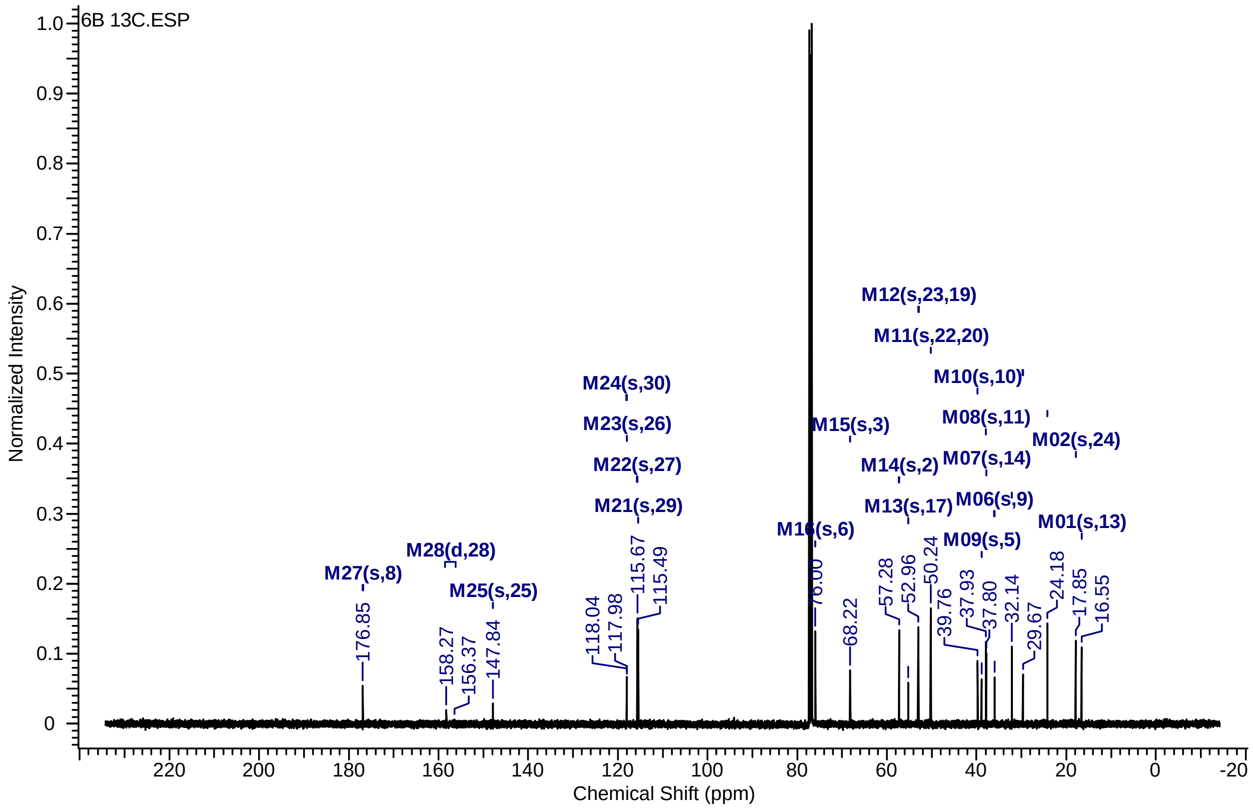

6B 13C.ESP
Normalized Intensity
Chemical Shift (ppm)
M27(s,8)
M28(d,28)
M25(s,25)
M24(s,30)
M23(s,26)
M22(s,27)
M21(s,29)
M16(s,6)
M15(s,3)
M14(s,2)
M13(s,17)
M12(s,23,19)
M11(s,22,20)
M10(s,10)
M08(s,11)
M07(s,14)
M06(s,9)
M09(s,5)
M02(s,24)
M01(s,13)
176.85
158.27
156.37
147.84
118.04
117.98
115.67
115.49
76.00
68.22
57.28
52.96
50.24
39.76
37.93
37.80
32.14
29.67
24.18
17.85
16.55

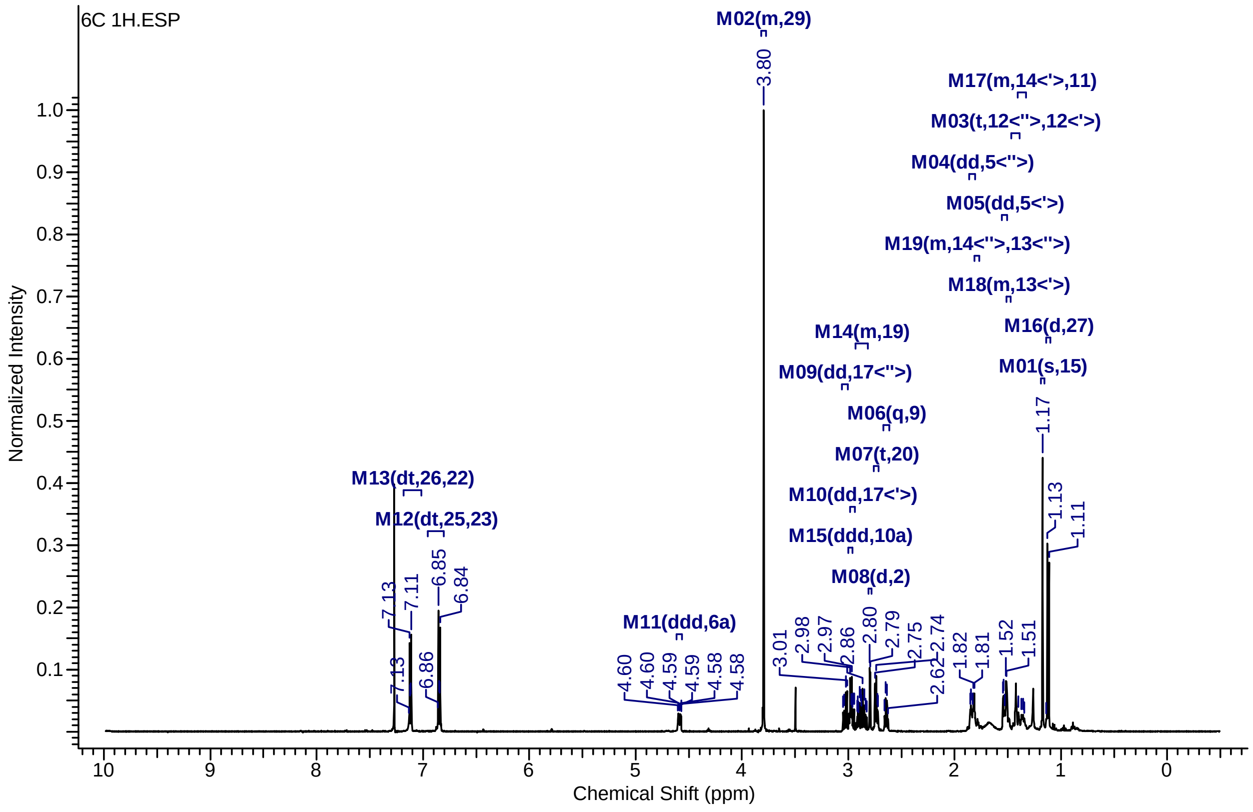
6C 1H.ESP
M02(m,29)
M17(m,14<'>,11)
M03(t,12<''>,12<'>)
M04(dd,5<''>)
M05(dd,5<'>)
M19(m,14<''>,13<''>)
M18(m,13<'>)
M14(m,19)
M16(d,27)
M09(dd,17<''>)
M01(s,15)
M06(q,9)
M07(t,20)
M13(dt,26,22)
M10(dd,17<'>)
M12(dt,25,23)
M15(ddd,10a)
M08(d,2)
M11(ddd,6a)
Normalized Intensity
Chemical Shift (ppm)

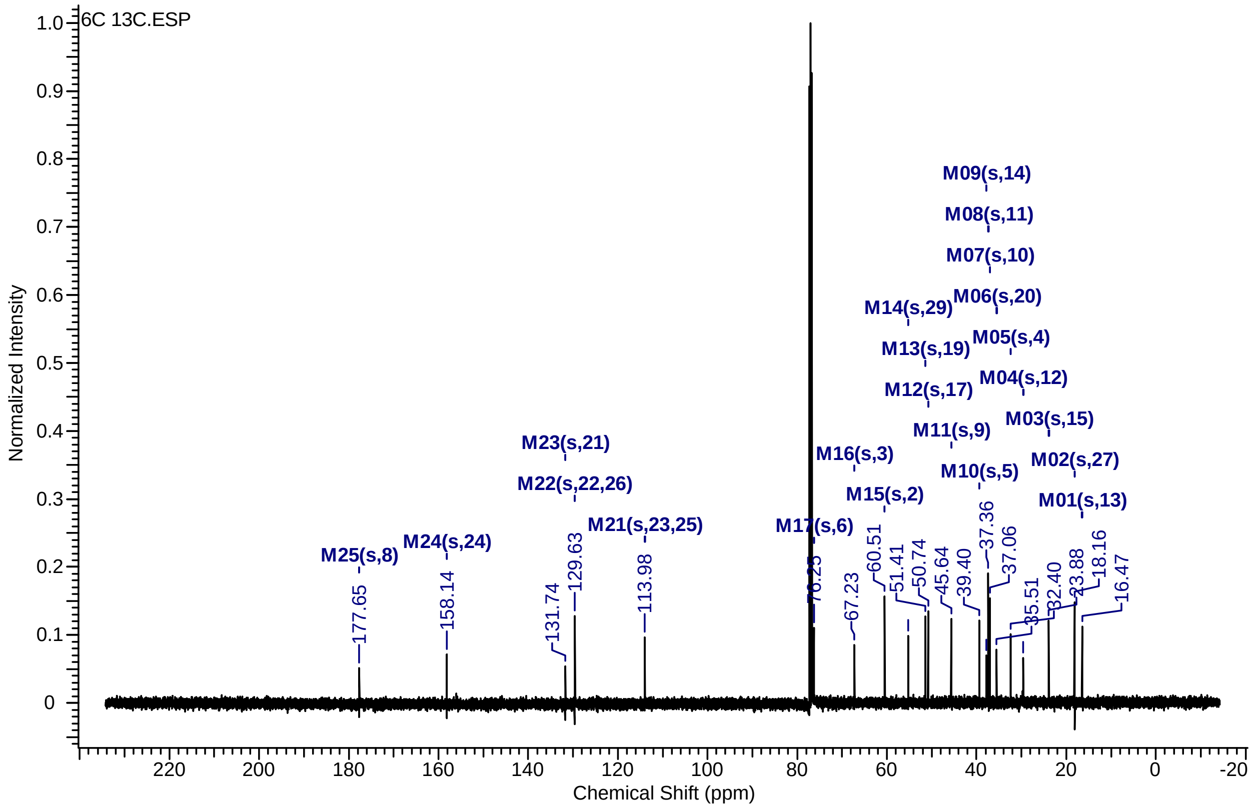
6C 13C.ESP
Normalized Intensity
Chemical Shift (ppm)
M25(s,8)
M24(s,24)
M23(s,21)
M22(s,22,26)
M21(s,23,25)
M17(s,6)
M16(s,3)
M15(s,2)
M14(s,29)
M13(s,19)
M12(s,17)
M11(s,9)
M10(s,5)
M09(s,14)
M08(s,11)
M07(s,10)
M06(s,20)
M05(s,4)
M04(s,12)
M03(s,15)
M02(s,27)
M01(s,13)
177.65
158.14
131.74
129.63
113.98
76.23
67.23
60.51
51.41
50.74
45.64
39.40
37.36
37.06
35.51
32.40
23.88
18.16
16.47

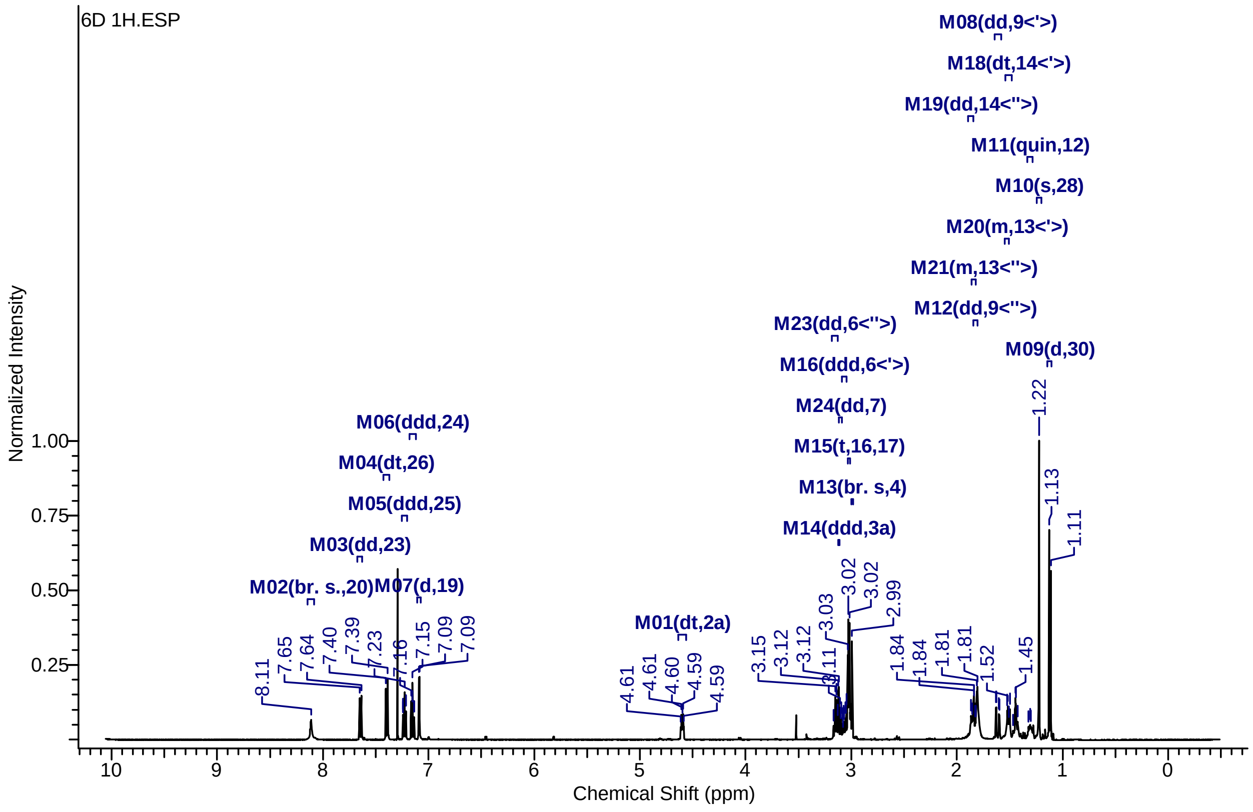

6D 1H.ESP
Normalized Intensity
Chemical Shift (ppm)
M01(dt,2a)
M02(br. s.,20)
M03(dd,23)
M04(dt,26)
M05(ddd,25)
M06(ddd,24)
M07(d,19)
M08(dd,9<'>)
M09(d,30)
M10(s,28)
M11(quin,12)
M12(dd,9<''>)
M13(br. s,4)
M14(ddd,3a)
M15(t,16,17)
M16(ddd,6<'>)
M18(dt,14<'>)
M19(dd,14<''>)
M20(m,13<'>)
M21(m,13<''>)
M23(dd,6<''>)
M24(dd,7)

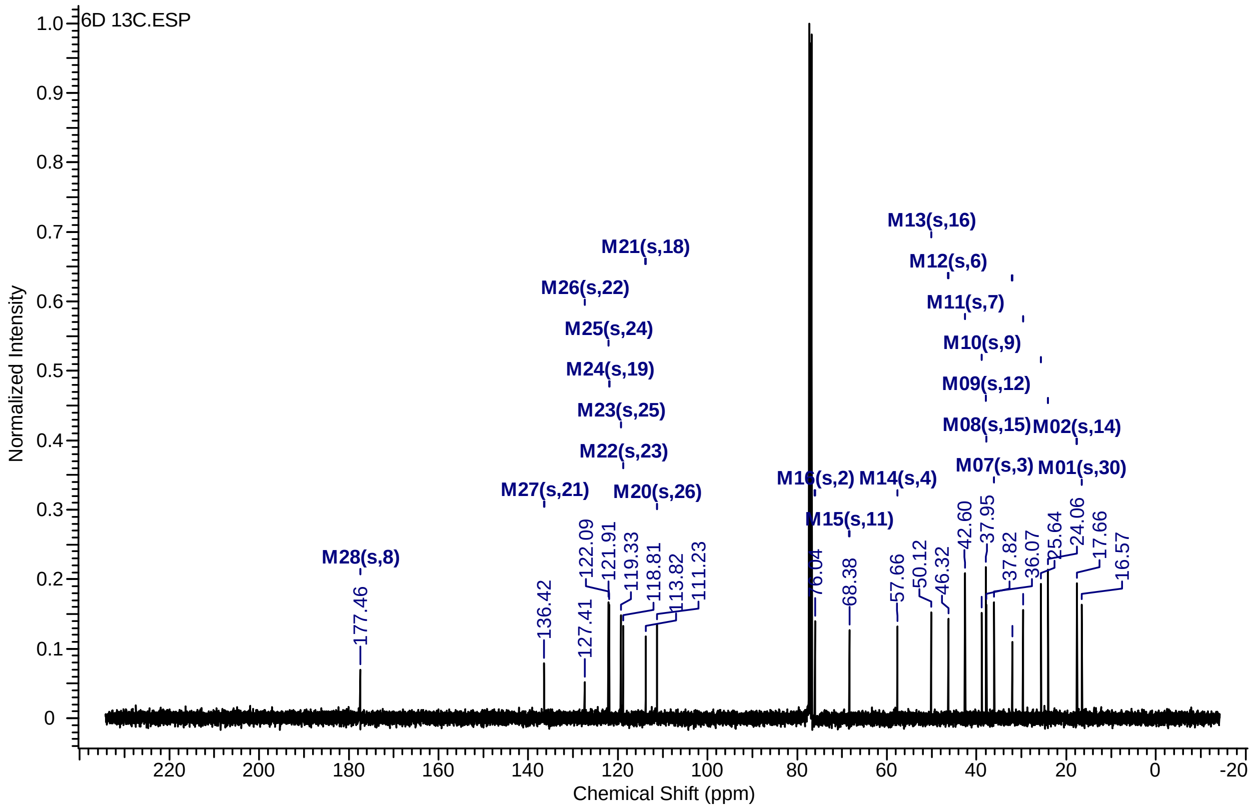

6D 13C.ESP
Normalized Intensity
Chemical Shift (ppm)
M28(s,8)
M27(s,21)
M26(s,22)
M25(s,24)
M24(s,19)
M23(s,25)
M22(s,23)
M21(s,18)
M20(s,26)
M16(s,2)
M15(s,11)
M14(s,4)
M13(s,16)
M12(s,6)
M11(s,7)
M10(s,9)
M09(s,12)
M08(s,15)
M07(s,3)
M02(s,14)
M01(s,30)
177.46
136.42
127.41
122.09
121.91
119.33
118.81
113.82
111.23
76.04
68.38
57.66
50.12
46.32
42.60
37.95
37.82
36.07
25.64
24.06
17.66
16.57
